# Exploring activity levels in physical education lessons in the UK: a cross-sectional examination of activity types and fitness levels

**DOI:** 10.1136/bmjsem-2020-000924

**Published:** 2021-03-09

**Authors:** Nick Beale, Emma Eldridge, Anne Delextrat, Patrick Esser, Oliver Bushnell, Emily Curtis, Thomas Wassenaar, Catherine Wheatley, Heidi Johansen-Berg, Helen Dawes

**Affiliations:** 1 Centre for Movement, Occupational and Rehabilitation Sciences (MOReS), Oxford Brookes University, Oxford, Oxfordshire, UK; 2 Wellcome Centre for Integrative Neuroimaging, FMRIB Centre, Nuffield Department of Clinical Neurosciences, John Radcliffe Hospital, University of Oxford, Oxford, Oxfordshire, UK; 3 NIHR Oxford Health Biomedical Research Centre, Oxford Health NHS Foundation Trust, Oxford, Oxfordshire, UK

**Keywords:** adolescent, secondary school, exercise testing, physical activity, aerobic fitness

## Abstract

**Objectives:**

To establish pupil fitness levels, and the relationship to global norms and physical education (PE) enjoyment. To measure and describe physical activity (PA) levels during secondary school PE lessons, in the context of recommended levels, and how levels vary with activity and lesson type.

**Methods:**

A cross-sectional design; 10 697 pupils aged 12.5 (SD 0.30) years; pupils who completed a multistage fitness test and wore accelerometers to measure PA during PE lessons. Multilevel models estimated fitness and PE activity levels, accounting for school and class-level clustering.

**Results:**

Cardiorespiratory fitness was higher in boys than girls (ß=−0.48; 95% CI −0.56 to −0.39, p<0.001), within absolute terms 51% of boys and 54% of girls above the 50th percentile of global norms. On average, pupils spent 23.8% of PE lessons in moderate-to-vigorous PA (MVPA), and 7.1% in vigorous PA (VPA). Fitness-focused lessons recorded most VPA in co-educational (ß=1.09; 95% CI 0.43 to 1.74) and boys-only lessons (ß=0.32; 95% CI −0.21 to 0.85). In girls-only lessons, track athletics recorded most VPA (ß=0.13; 95% CI −0.50 to 0.75) and net/wall/racket games (ß=0.97; 95% CI 0.12 to 1.82) the most MVPA. For all lesson types, field athletics was least active (ß=−0.85; 95% CI −1.33 to −0.36). There was a relationship of enjoyment of PE to fitness (ß=1.03; 95% CI 0.83 to 1.23), and this relationship did not vary with sex (ß=−0.14 to 0.23; 95% CI −0.16 to 0.60).

**Conclusions:**

PE lessons were inactive compared with current guidelines. We propose that if we are to continue to develop a range of sporting skills in schools at the same time as increasing levels of fitness and PA, there is a need to introduce additional sessions of PE activity focused on increasing physical activity.

**Trial registration number:**

NCT03286725.

**What are the new findings?**

- Clear benchmarks against guidelines from a large-scale representative study for cardiorespiratory fitness levels of pupils in Year 7, and activity-specific levels of physical activity (PA) intensities in physical education (PE) lessons.
- PE lessons were inactive compared with current guidelines; choice of activity in combination with lesson type (sex composition) and enjoyment relate to PA levels.
- There is a clear hierarchy of PE activities, with some differences recorded for moderate-to-vigorous PA (MVPA) and girls-only lessons; however there are more similarities between groups, particularly enjoyment of PE, where regardless of sex, pupils that ‘strongly agreed’ to enjoying PE were fitter than their counterparts.

**How might it impact on practice in the future?**

- If increasing cardiorespiratory fitness and PA levels of pupils is an objective, then teachers must consider both how to maximise enjoyment of PE alongside introducing more MVPA sessions in school.
- Recommendations for teachers to deliver higher PA in PE, and reduce sedentary time. This might be through choice of activity, lesson type, or the inclusion of new elements to a lesson to both increase activity intensity and enjoyment.
- Certain types of PE activity are less active and it may be that, in order to continue to develop a range of sporting skills and achieve higher levels of PA in children for health and well-being, we may need to adopt a novel approach with the introduction of additional fitness-type sessions within schools alongside standard PE where sporting skills are built.

## Introduction

Cardiorespiratory fitness (CRF) is related to better physical and psychological health^1^ and higher academic achievement in schoolchildren,^2–4^ with higher childhood fitness being linked to better health, well-being and life-chances in adulthood.^5 6^ Adolescent fitness levels have been falling globally,^7–10^ raising concerns regarding the long-term impact. Alongside non-modifiable biological and genetic factors,^11^ physical activity (PA) is a key modifiable determinant of fitness.^12^ Indeed, moderate-to-vigorous PA (MVPA) levels in childhood are known to be critical for the healthy development of metabolic, cardiovascular and musculoskeletal systems. Activity levels decline throughout adolescence,^13^ particularly in girls, and those who are more socioeconomically disadvantaged, or living in inner-city areas.^14–16^ Worryingly just 43.2% of adolescents in the UK now meet the current government activity guidelines, which suggest accumulating at least 60 min MVPA per day across the week.^17–19^

Most young people in the UK have to attend school, and physical education (PE) lessons are compulsory until Year 11,^13^ suggesting that school PE offers a suitable setting to promote adolescent PA and fitness.^20 21^ During the past decade, focus on PE has shifted from fitness and competition^22 23^ to learning experiences, skills development and fostering the benefits of regular PA.^24^ The UK Association for Physical Education recommends pupils should be actively moving for 50%–80% of the available PE lesson time,^25^ although no intensity level is specified. Previous studies suggest pupils spend an average of 40.5% of PE lesson time in MVPA.^26^ Notably, time spent being sedentary or performing light activities in lesson time has been less clearly reported, but one Japanese study showed primary schoolchildren not moving for 27.3% of the time in PE.^27^ Thus, provisional data suggest that a great deal of PE time might be spent standing or sitting and that lessons could be adapted to increase activity levels.

When considering factors affecting PA, a recent systematic review^28^ identified modifiable variables that were consistently associated with levels of MVPA in PE including the class sex, the type of PE activities and content, lesson location (outdoors), beliefs and values of students, and enjoyment of exercise. The current levels of fitness and PA in PE in the UK are not well described; there are no recent large-scale surveys of PA in English PE lessons using accelerometry. A clear benchmark of performance against guidelines, from a large-scale representative study, is required to inform future policy. Our aim was to describe fitness, PA levels and patterns of PA in PE lessons alongside measuring factors known to affect activity levels. Our primary objectives were to describe: (1) the CRF levels of Year 7 pupils by sex in relation to global norms and enjoyment of PE, (2) the levels of sedentary PA (SPA), MVPA and vigorous PA (VPA) in PE lessons in the context of recommended levels, (3) the effect of activity type and lesson type (sex composition), in combination, on activity levels in PE.

## Methods

**Figure 1:**
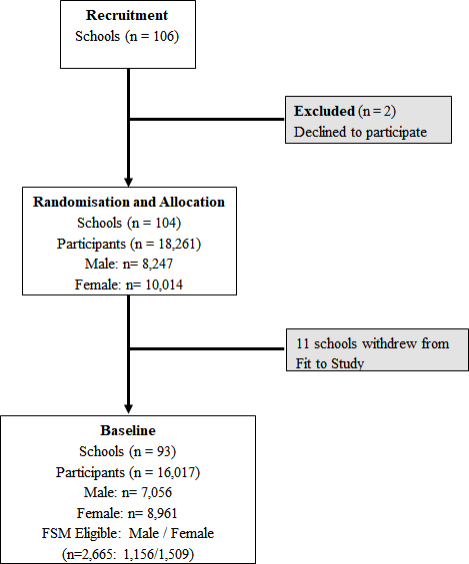
School recruitment and participant flow chart for the ‘Fit to Study’ project’s baseline data. FSM, free school meal.

We used a subsample of baseline data from the ‘Fit to Study’ cluster-randomised controlled trial—10 697 pupils aged 12.5 (SD 0.30) years. Figure 1 presents a flow chart for school and participant recruitment for the collection of baseline data (16 017 pupils). Details of the trial, including recruitment, methodology and consent procedures, are reported in the study protocol.^29^ Baseline data for each measure of interest are presented in online supplemental file 1. Primary analyses included participants who completed each measure of interest at baseline (online supplemental file 2).

10.1136/bmjsem-2020-000924.supp1 Supplementary data

10.1136/bmjsem-2020-000924.supp2 Supplementary data

### Participants and setting

Participants were pupils aged 11–13 years from the UK state secondary schools. Baseline assessments were undertaken between June and September 2017, at the end of Year 7 and the start of Year 8. Schools provided participants’ sex, birth date and pupil eligibility for free school meals (eFSMs), an indicator of socioeconomic deprivation. Participants completed questionnaires on school computers, or otherwise at home. Additional information on measures and data cleaning procedures are reported in online supplemental file 2.

### Outcome measures

CRF was assessed by PE teachers during normal PE lessons, using a standardised multistage 20-metre shuttle run test.^30^ Total number of laps completed was recorded: we compared pupils’ performances with normative 50th percentile scores by sex aged 12 years.^31^ We performed concurrent validity testing on the shuttle run test, through comparison of the field-based and lab-based (a cardiopulmonary exercise test) fitness measures in a subsample who participated in a brain imaging substudy (online supplemental file 3).

10.1136/bmjsem-2020-000924.supp3 Supplementary data

Pupil PA during PE lessons was measured with wrist-worn AX3 triaxial accelerometers designed by Open Lab, Newcastle University.^32^ Through visits to a single lesson, we aimed to measure at least half of the year group in every school, which in some cases required multiple visits. All pupils in a lesson wore a monitor, excluding those who had opted out of the study. Pupils were not identified individually. A member of the research team noted the number of pupils per class, and the type of activity with reference to a General Certificate of Secondary Education (GCSE) classification of sports families.^33^ Whether lessons were single sex or mixed sex, and whether they took place indoors or outdoors was also noted. To describe PA patterns, we calculated class average minutes of SPA, light PA, moderate PA and VPA for the ‘effective’ lesson (timetabled lesson time minus changing time) and standardised this value to minutes per hour to account for different lesson lengths. The raw accelerometry data were processed into PA ‘counts’ using a 1 s epoch^34–38^ and based on established ‘cut-off points’.^39^ Further detail is provided in online supplemental file 2.

PE enjoyment was measured with a single item, ‘I enjoy PE’ (1=‘strongly disagree’ to 7=‘strongly agree’) via an online questionnaire.^40–42^

### Statistical analyses

Demographic data were analysed using descriptive statistics. Multilevel modelling was used to estimate the fitness levels of pupils, and the activity levels in PE, accounting for school and class-level clustering. Full details of model development and specification, data transformations and sensitivity analyses are reported in online supplemental file 4. All analysis was performed in R V.3.5.3,^43^ using linear mixed-effects analysis. Pairwise comparisons for the fixed factors, where model estimates indicated significance, were examined as differences of least squares means adjusted according to Tukey.

10.1136/bmjsem-2020-000924.supp4 Supplementary data

### Patient and public involvement

The ‘Fit to Study’ project (http://www.fit-to-study.org/) included an 18-month participatory and co-design development phase to establish and refine the measurement approaches. This included consultation with national and local sports associations, and PE teachers from eight local secondary schools, and guidance from a project Steering Advisory Group. Plans for recruitment were developed with the funders. No parties outside the research team were involved in implementation of the study, or were asked to advise on interpretation or writing up of results.

## Results

### Demographic data

Demographic data are provided in online supplemental file 1. Mean age at the start of the school year was 12.5 (SD 0.30) years. The total number of lessons visited and pupils participating in these lessons is summarised (for activity group and lesson type) in table 1. A summary for all school-level and lesson-level variables is provided in online supplemental files 5 and 6. After data cleaning, 10 697 participants (girls=6078; 57%; eFSM=1647; 15.4%) from 74 schools completed the fitness test. Of these, 7485 (girls=4495; 60%; eFSM=1071; 14.3%) from 67 schools also completed the questionnaire. A total of 9483 participants (not individually identified) from 88 schools had their PA levels monitored during 249 PE lessons.

10.1136/bmjsem-2020-000924.supp5 Supplementary data

10.1136/bmjsem-2020-000924.supp6 Supplementary data

**Table 1:** Number of lessons visited (A) and number of participating pupils (B) by activity group and by lesson type

Activity group		Lesson type			
	A B	Girls (n=60) (n=1961)	Boys (n=86) (n=2446)	Mixed (n=103) (n=5076)	Overall (n=249) (n=9483)
Invasion games	A B	13 (21.7%) 328 (16.7%)	19 (22.1%) 621 (25.4%)	5 (4.9%) 136 (2.7%)	37 (14.9%) 1085 (11.4%)
Net/wall/racket games	A B	3 (5.0%) 73 (3.7%)	8 (9.3%) 191 (7.8%)	5 (4.9%) 130 (2.6%)	16 (6.4%) 394 (4.2%)
Fielding/striking games	A B	17 (28.3%) 601 (30.6%)	37 (43.0%) 1058 (43.3%)	33 (32.0%) 1253 (24.7%)	87 (34.9%) 2912 (30.7%)
Athletics	A B	5 (8.3%) 110 (5.6%)	4 (4.7%) 105 (4.3%)	3 (2.9%) 114 (2.2%)	12 (4.8%) 329 (3.5%)
Fitness	A B	4 (6.7%) 88 (4.5%)	8 (9.3%) 243 (9.9%)	4 (3.9%) 170 (3.3%)	16 (6.4%) 501 (5.3%)
Adventure/games	A B	1 (1.7%) 16 (0.8%)	2 (2.3%) 55 (2.2%)	1 (1.0%) 41 (0.8%)	4 (1.6%) 112 (1.2%)
Various	A B	14 (23.3%) 661 (33.7%)	1 (1.2%) 20 (0.8%)	41 (39.8%) 2882 (56.8%)	56 (22.5%) 3563 (37.6%)
Athletics—field	A B	3 (5.0%) 84 (4.3%)	5 (5.8%) 93 (3.8%)	5 (4.9%) 135 (2.7%)	13 (5.2%) 312 (3.3%)
Athletics—track	A B	0 (0%) 0 (0%)	2 (2.3%) 60 (2.5%)	6 (5.8%) 215 (4.2%)	8 (3.2%) 275 (2.9%)

### Fitness descriptives in comparison with global normal values

The mean absolute fitness levels and comparison with global norms are presented in table 2.

**Table 2:** Fitness (cumulative laps) for (A) sex, (B) eFSM, (C) sex by eFSM, compared with global norms (gn)

		Mean (SD)	Q_2_ laps	Q_2_ gn laps	n (%) above gn
A: sex p<0.001*	Boys (n=4619) Girls (n=6078) Total (n=10 697)	43.8 (23.5) 32.7 (16.7) 37.5 (20.7)	40 29 33	39 28 –	2365 (51) 3300 (54) 5665 (53)
B: eFSM p<0.001	No (n=9050) Yes (n=1647)	38.5 (20.9) 32.0 (18.4)	34 28		
		Mean (SD)	n (%) above gn		
C: sex by eFSM	Boys: No (n=3913) Yes (n=706)	44.9 (23.7) 37.5 (21.6)	2069 (53) 296 (42)		
p=0.469	Girls: No (n=5137) Yes (n=941)	33.6 (16.9) 27.8 (14.2)	2887 (56) 413 (44)		

Q_2_=50th percentile.

*The p value is a simple approximation, based on the t-statistics and using the normal distribution function.

eFSM, eligibility for free school meal.

The results of the concurrent validity testing of in-school assessment compared with laboratory VO_2_ max testing are presented in online supplemental file 3. Only one data point lied outside the 95% limits.

### PE enjoyment

**Figure 2:**
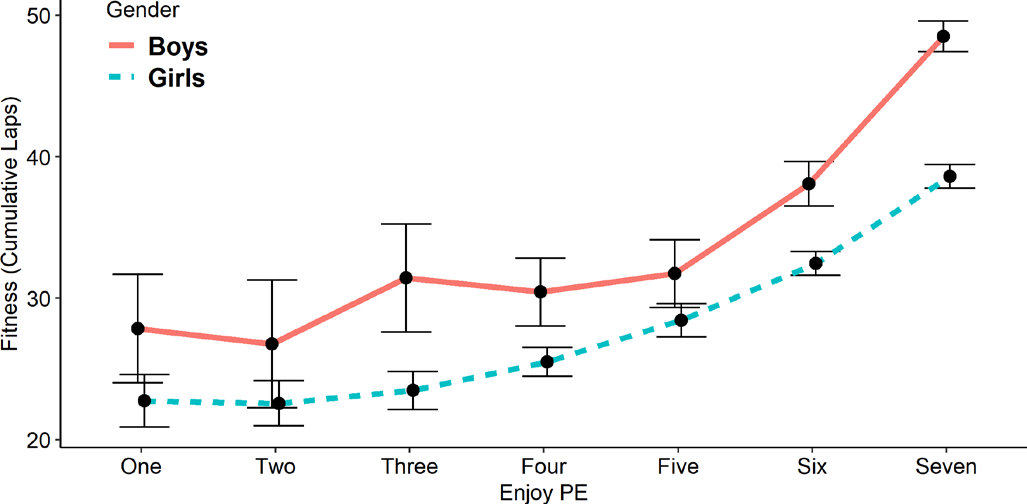
Grouped line plot of fitness (cumulative laps) by physical education (PE) enjoyment, by sex.

The aggregated results are plotted as a line graph of fitness to the ‘PE enjoyment’ measure by sex (figure 2). A multilevel model was used to investigate the effect of PE enjoyment on fitness according to sex. The results (online supplemental file 4) showed that fitness was positively related to PE enjoyment (ß=1.03; 95% CI 0.83 to 1.23). Moreover, the relationship was stronger among boys than girls (ß=−0.28; 95% CI −0.52 to −0.04).

### Multilevel models of fitness levels

Online supplemental file 7 summarises associations between fitness and predictor variables. In fully adjusted models, 15.1% of the variance was explained by school.

10.1136/bmjsem-2020-000924.supp7 Supplementary data

The primary aim was to show how CRF varied between sex, accounting for differences between schools. The results (online supplemental file 7, Model 1 estimate) showed fitness varied significantly between boys and girls (ß=−0.48; p<0.001), and between eFSM pupils and their counterparts (ß=−0.22; p<0.001). There was no significant interaction effect between sex and FSM status in terms of their relationship with CRF.

Second, we explored how fitness varied between school location (based on postcode Index of Multiple Deprivation (IMD)^44^ tertiles), and the interaction of school type (co-educational or single sex). The results (online supplemental file 7, Model 2 estimate) showed that pupils in schools located in areas of low deprivation recorded higher levels of fitness compared with pupils in schools located in areas of high deprivation (ß=0.40; p<0.013). There was no significant main effect of school type, and there was no significant interaction effect between IMD tertile and school type in terms of their relationships with fitness. There was also no significant difference in girls’ fitness between girls educated in co-educated schools compared with girls-only schools (online supplemental file 7, Model 3 estimate).

### PE lesson PA descriptives

**Figure 3:**
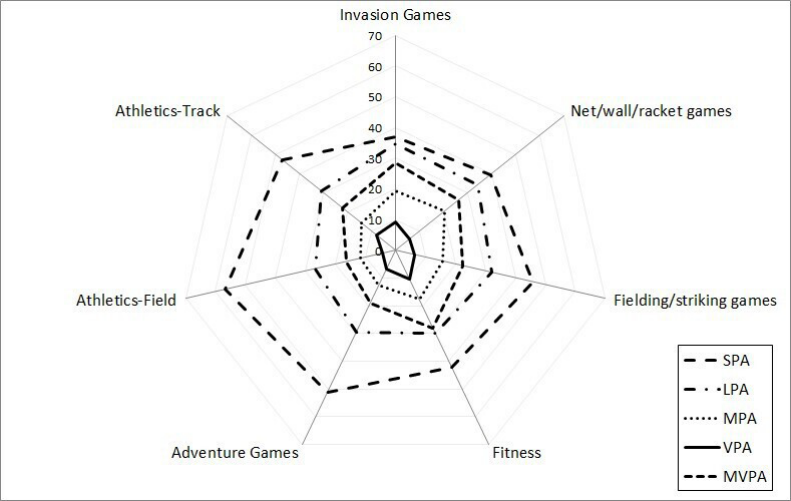
Average activity level (% of lesson) across physical activity (PA) domains, by activity group. LPA, light PA; MPA, moderate PA; MVPA, moderate-to-vigorous PA; SPA, sedentary PA; VPA, vigorous PA.

In summary, on average across schools, 23.7% of the time was spent in MVPA, 7.0% in VPA and 44.3% in SPA, respectively; table 3 and figure 3 present PA recorded for each type of activity. On average, the ‘effective’ or actual lesson time was 75.3% of the timetabled lesson (online supplemental file 5). The mean (SD) lesson-level PA during PE, expressed as a percentage of the lesson, for all PA domains is presented in online supplemental file 8 (for school location/type and lesson type) and in online supplemental file 9 (for activity group).

10.1136/bmjsem-2020-000924.supp8 Supplementary data

10.1136/bmjsem-2020-000924.supp9 Supplementary data

**Table 3:** Percentage of lesson time spent in physical activity (PA) domains, and the percentage of pupils achieving PA thresholds, by activity group

Activity group (ordered by VPA)	% of lesson			% of pupils meeting/hour		
	VPA	MVPA	SPA	>5 min VPA	>30 min MVPA	<20 min SPA
Fitness	10.2	27.9	42.1	55.9	0.4	22.0
Invasion games	9.2	28.4	37.0	48.4	2.1	40.6
Track athletics	7.9	22.1	47.0	40.4	0.4	10.6
Adventure games	6.7	19.1	51.3	37.5	0.0	16.1
Fielding/striking games	6.5	22.3	45.8	26.9	0.3	13.8
Net/wall/racket games	5.8	26.2	39.5	21.1	1.8	33.5
Field athletics	4.5	16.3	56.8	14.4	0.0	3.5

MPA, moderate PA; MVPA, moderate-to-vigorous PA; PA, physical activity; SPA, sedentary PA; VPA, vigorous PA.

For MVPA and VPA, co-educational schools recorded higher levels of activity compared with single-sex schools (24.2% vs 21.6%, and 7.3% vs 5.6%, respectively), and schools located in areas of high deprivation recorded lower activity levels (22.7% vs 24.2%, and 6.5% vs 7.4%, respectively) than schools located in areas of low deprivation. Boys-only lessons were the most active (24.6% and 7.7%), followed by mixed lessons (23.8% and 6.9%) and then girls-only lessons (22.4% and 6.2%).

**Figure 4:**
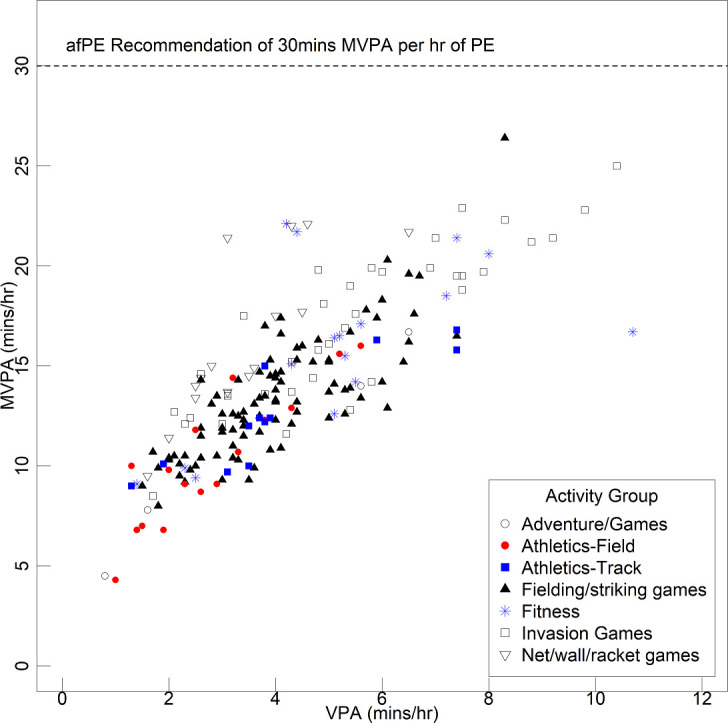
Grouped scatter of lesson average MVPA by lesson average VPA, by activity group. afPE, Association for Physical Education; PE, physical education; MVPA, moderate-to-vigorous physical activity; VPA, vigorous physical activity.

Figure 4 presents the relationship between lesson-average MVPA and VPA, by activity group, with no lesson achieving 30 min MVPA per hour of PE. The most active lessons by VPA were fitness and invasion games, with field athletics the least active. It was similar for MVPA, although the top-ranked single lesson for this intensity level was fielding/striking games. Violin plots of pupil average time (minutes/hour) split by PA intensity domains and activity group are presented in online supplemental file 9.

### Multilevel models of PA levels during PE

Our primary objective was to describe the effect of activity type and lesson type on PA levels during PE classes, and how levels varied with different combinations of these predictors. Online supplemental files 10-12 summarise associations between PA levels and predictor variables.

10.1136/bmjsem-2020-000924.supp10 Supplementary data

10.1136/bmjsem-2020-000924.supp11 Supplementary data

10.1136/bmjsem-2020-000924.supp12 Supplementary data

The results for school-level predictor variables (online supplemental file 10, Table 1 | Model 1 estimates) showed no significant effects of school type (ß=−0.18 to 0.05; p=0.421–0.911), school FSM status (ß=−0.02 to 0.02; p=0.916–0.938) or lesson type (ß=−0.21 to 0.25; p=0.246–0.975) on activity levels in PE. There was no significant interaction effect between school FSM and lesson type in terms of their effects on activity levels (ß=−0.15 to 0.18; p=0.445–0.998). Boys-only lessons were the most vigorously active (ß=0.23; p=0.338), with mixed lessons the least active (ß=−0.08; p=0.752). Girls-only lessons were the most sedentary (ß=0.06; p=0.826) and the least active in terms of MVPA (ß=0.04; p=0.877).

When considering the main effect of activity group, classes explained more variance than schools. School cluster effects explained 10.6%, 6.8% and 6.3% of the variance in VPA, MVPA and SPA, whereas class effects explained 22.1%, 20.8% and 21.9%, respectively. The results (online supplemental file 10, Table 2 | Model 2 estimates) showed fitness lessons and track athletics were characterised by the highest levels of activity (highest positive ß values), with fielding/striking games and field athletics showing the lowest activity levels, consistent with trends visible in figure 3. Post-hoc analysis directly comparing activity types (online supplemental file 12, Model 2—VPA, MVPA and SPA) reinforced these patterns.

When investigating the effect of ‘lesson type’, the results (online supplemental file 10, Table 3 | Model 3 estimates), showed boys-only classes were the most active (highest positive ß values), while mixed and girls-only classes exhibited similar activity levels (near to zero ß values). Mixed and girls-only classes were also the most sedentary. No significant main effect was observed, however, a significant interaction effect of ‘activity group by lesson type’ was observed for all the PA domains modelled (online supplemental file 10, Table 3 | Model 3 estimates).

For both boys’ and mixed lessons, fitness-focused lessons recorded the highest levels of VPA (online supplemental file 11, Table 2 | Model 3—VPA). For girls-only lessons, fitness recorded the lowest VPA levels of all activity groups, with track athletics the highest. The lowest levels of activity in boys’ and mixed lessons were recorded for field athletics. For girls’ lessons, net/wall/racket games had the highest levels of MVPA (online supplemental file 11, Table 4 | Model 3–MVPA). For all lesson types, field athletics was the most sedentary (online supplemental file 11, Table 6 | Model 3–SPA); fitness being the least sedentary for boys’ and mixed lessons, and net/wall/racket being the least sedentary for girls' lessons (online supplemental file 11, Table 6 | Model 3–SPA). Post-hoc analysis (online supplemental file 12, Model 3) reinforced these patterns.

## Discussion

We observed that the fitness levels of Year 7 schoolchildren in the UK were similar to current global norms (table 2). Girls were less fit than boys as were young people from lower socioeconomic backgrounds or from schools located in more deprived areas. We also noted that activity levels in PE lessons were low compared with guidelines,^25^ and low compared with the most recent meta-analysis of global levels of PA in PE.^26^ Less than 1% of pupils achieving the suggested level of activity, and an overall lesson average MVPA level of only 23.8% compared with the recommended 50%–80% of lesson time. Of note, the most commonly observed lesson activity—fielding/striking games—was one of the least active, along with field athletics. Some PE activities, particularly fitness, invasion games and track athletics, were more active—though still below guideline levels for MVPA. Once the type of activity was taken into account, there was no difference between single-sex and co-educational lessons in MVPA. Taken together our findings suggest the need for a novel approach to meet the need to develop a wide range of sporting skills and increase physical activity and fitness in school PE by possibly introducing separate fitness sessions.

### Current fitness levels of young UK adolescents

In comparison with current global normative values,^31^ for both boys and girls, we found average CRF was marginally higher with 51% of boys, and 54% of girls above the 50th percentile. Our findings should be considered alongside the known decline in fitness in recent decades, with annual declines ranging from 0.43%^7 31^ to 1%^10 45^ between 1998 and 2014, although now stabilised.^31^ Our results also confirm lower fitness compared with global norms for lower socioeconomic status (SES) pupils, in both boys (42%) and girls (44%). By contrast a US study,^46^ that observed a sample of 954 urban middle school pupils, found that SES was related to physical fitness only in girls. Finally, we observed that 15.1% of variance was explained by school effects. This might in part reflect the influence of school location, with pupils in schools located in areas of low deprivation recording higher levels of fitness compared with pupils in schools located in the highest deprived tertile.

### Current levels of PA during secondary school PE lessons

Our findings show that pupils are not very active in PE classes, as not a single lesson achieved 30 min MVPA per hour of PE. The lesson average MVPA was only 23.8%, and sedentary time was 44.3%, and only 73 of 9483 pupils monitored achieved the 30 min MVPA threshold. Levels are well below the 40.5% identified in the meta-analysis by Hollis *et al*,^26^ although this review covered nine countries including the UK, a mix of observational and objective data, and varied protocols and assumptions so is not fully representative of the UK position, unlike our study. An earlier review of British lessons reported this figure to be between 27% and 47%, and highlighted a large interindividual difference in MVPA levels across pupils.^47 48^ The ‘Fit to Study’ pilot study (Delextrat, 2019) recorded an average figure of 30.7% MVPA when considering ‘effective’ PE time.^49^

It has been suggested that a pursuit of PA alone may result in teachers prioritising fitness-based activities, at the expense of enjoyment and developing physically literacy.^50^ However, our observations during the summer curriculum was that fitness-based activities were not prioritised, and that fielding and striking games lessons, one of the least active lesson types, were most commonly observed. Some PE activities, particularly fitness, invasion games and track athletics, were the most active—though even for these more active lessons, only around 22%–28% of lesson time was spent in MVPA, and 8%–10% spent in VPA. Sedentary behaviour was less evident in invasion games and net/wall rackets games, with over 40% and 33% of pupils, respectively, exhibiting less than 20 min SPA per hour of PE in these activities.

We found no significant differences between boys, girls and mixed-sex lessons. This is in contrast to some past studies^51–54^ that reported girls were more physically active in mixed-sex classes compared with girls-only classes, and that boys engage in more VPA and MVPA than girls^55–59^ depending on the type of activity.^55 60–65^ Our findings may be more robust as we were able to take account of school/class-level clustering. On the other hand, we did not aim to evenly sample mixed and single-sex lessons and so our sample is unbalanced in terms of lesson sex composition. Our results showed a significant interaction effect between activity type and sex of lessons for all intensity levels. For both boys’ and mixed lessons, fitness recorded the highest levels of VPA and field athletics the lowest. For girls-only lessons, fitness recorded the lowest VPA levels, with track athletics the highest, and net/wall/racket games the highest levels in terms of MVPA.

We showed that pupils who ‘strongly agreed’ to enjoying PE were fitter than their counterparts, and that this relationship did not vary with sex (figure 2, online supplemental file 4). Further work would be required to confirm directionality, but looking at this result in isolation could indicate that PE should prioritise the joy of exercise and movement over high intensity. However, given the current low levels of intense activity we measured in PE, and the positive relationship between higher intensity and improved fitness, both aspects could be equally important and should be considered in future lesson planning.

### Strengths and limitations

Our main strength was a large objectively measured sample which allowed for a hierarchical structure to the data analysis and better understanding of factors affecting PA in PE. The analysis was cross-sectional, and cannot determine cause and effect, and while effort was made to achieve a representative sample this may not have been achieved. Unfortunately, we were unable to explore the effect of individual pupil sex on PA levels during PE sessions, as pupils in PE lessons were not individually identified, and individual activity levels in PE could not be matched to other measures.

With our large sample size, all fully nested random structure models converged, with all fixed-effect terms of interest included. However, it should be noted that the dataset could have included a better representation (balance) of each subtype of independent variable in each class/school, and consequently the imbalance of observed number of PE activities might have introduced bias due to multiple comparisons and small power. However, this cannot be forced if the aim of the study is to examine current practices without intervening. A lack of a fully balanced dataset and the fact that we only recorded PA during one PE class per pupil should also be acknowledged as potential confounding factors.

We objectively measured PA and fitness which adds to previous understanding garnered via subjective means; adolescents often perceive themselves to be more physically active than they actually are,^66^ which can provide misleading results. However, there is some indication in our sample that a higher number of eFSM boys and girls did not participate in the fitness test, raising the possibility that the sample is not fully representative. The positioning of accelerometers for measuring PA levels may affect activity recordings.^67^ However, our methodology is in agreement with other large cohort studies, which derived similar parameters in the general population.^32^ It is also possible that our choice of epoch times and cut-off points has influenced results,^60^ and a researcher being present during testing in PE lessons might also have had an effect on teacher performance and pupil activity levels. Other factors that could influence PA levels were not recorded, for example, the state and size of school PE facilities and resources, including the number of PE staff, as well as the impact of weather conditions during testing that would have dictated location and choice of some activities.

Finally, we observed that pupils who were fitter were more likely to enjoy PE. While we were unable to explore enjoyment in relation to objective PA levels during PE (as we did not record pupil identity for accelerometry measures), it has previously been reported that pupils who report enjoying PE more engage in greater physical activity outside of school,^68^ and are likely to be fitter as a consequence.

## Recommendations

Considering that school for many young people is the main opportunity for being physically active, our results support the ideas expressed in the UK Government 2019 School Sport and Activity Action Plan,^69^ that PE lessons cannot bear the whole burden of delivering PA and fitness. However, we suggest that if teachers are attempting to deliver more active PE, we recommend they take into consideration activity choice and the impact of sex composition of classes. We suggest that regardless of location, invasion games, track athletics and fitness lessons will provide an opportunity for higher levels of VPA, and that teachers may wish to include fitness infusions during less active lessons such as field athletics, or when teaching ‘skills’ is a focus of the lesson, as there is evidence that short bouts of VPA can improve adolescent fitness.^70 71^

If increasing CRF levels of pupils is also an objective, then teachers should consider enjoyment of PE alongside introducing more highly intense activities. The inter-relationship should be examined in interventional studies. It may be that additional fitness sessions need to be introduced in schools in order to address the health and well-being needs of young people.

Finally, and supporting past recommendations,^31 72^ the monitoring of PA levels in PE and the fitness of all pupils, especially the least fit children in deprived areas, should be considered as part of any future activity action plan or exercise intervention in schools.

10.1136/bmjsem-2020-000924.supp13 Supplementary data
