# Supplementary material for: Exploring activity levels in physical education lessons in the UK: a cross-sectional examination of activity types and fitness levels
Source: BMJ Open Sport Exerc Med. 2021 Mar 9;7(1):e000924. doi: 10.1136/bmjsem-2020-000924 (PMC7944978; doi:10.1136/bmjsem-2020-000924)
Supplement: Supplementary data [file bmjsem-2020-000924supp001.pdf]

Exploring activity levels in physical education lessons in the UK: A cross-sectional examination of activity types and fitness levels

SUPPLEMENTARY FILE 1: Fit-to-Study baseline demographic data

Table: Demographic Data for Schools/Pupils that participated in the 'Fit to Study' project and completed baseline assessments

		**Full Baseline Dataset**			**Baseline Fitness Dataset**			**Baseline Questionnaire Dataset**			**Baseline PE Lesson Dataset**
		**Male**	**Female**	**Total**	**Male**	**Female**	**Total**	**Male**	**Female**	**Total**	
School Type (School Data)	Co-Ed	-	-	75	-	-	59	-	-	52	70 (206)
	Female	-	-	17	-	-	14	-	-	14	17 (42)
	Male	-	-	1	-	-	1	-	-	1	1 (1)
	Total (Classes)	-	-	93	-	-	74	-	-	67	88 (249)
School Type (Pupil Data)	Co-Ed	6993	6294	13287	4563	4167	8730	2955	2906	5861	8059
	Female	0	2667	2667	0	1911	1911	0	1589	1589	1408
	Male	63	0	63	56	0	56	35	0	35	16
	Total (%)	7056 (44)	8961 (56)	16017	4619 (43)	6078 (57)	10697	2990 (40)	4495 (60)	7485	9483
Free School Meals Eligible	No	5900	7452	13352	3913	5137	9050	2579	3835	6414	-
	Yes (%)	1156 (16.4)	1509 (16.8)	2665 (16.6)	706 (15.3)	941 (15.5)	1647 (15.4)	411 (13.7)	660 (14.7)	1071 (14.3)	-
Term Completed Test	Summer 2017	-	-	-	3795	5235	9030	-	-	-	-
	Autumn 2017	-	-	-	824	843	1667	-	-	-	-
PE Visits (No of lessons)	Summer 2017	-	-	-	-	-	-	-	-	-	100 (214)
	Autumn 2017	-	-	-	-	-	-	-	-	-	11 (35)
Questionnaire Completed	Term time	-	-	-	-	-	-	2,831	4,295	7,090	-
	Holidays	-	-	-	-	-	-	159	236	395	-
Age, Years (as at 1 Sept. 2017)	Mean (SD)	12.49 (0.29)	12.50 (0.30)	12.49 (0.29)	12.49 (0.29)	12.50 (0.30)	12.49 (0.30)	12.49 (0.29)	12.50 (0.29)	12.50 (0.29)	-
	Min-Max	11.76-13.88	11.60-14.66	11.60-14.66	12.00-13.88	12.00-14.66	12.00-14.66	12.00-13.73	12.00-13.96	12.00-13.96	-

Exploring activity levels in physical education lessons in the UK: A cross-sectional examination of activity types and fitness levels

Table Notes

- PE lesson fitness test results were received from 74 schools, covering 10,697 / 12,534 pupils (85.3%).
- PE lesson physical activity data was collected in 88 schools, in June-July 2017 (100 visits) and September-October 2017 (11 visits). A total of 249 PE classes, and 9,483 pupils, were covered. This represented, on average, 60.5% of total pupils in the 88 schools. The aim was to capture at least 50% of the pupils in the year group, either through a single visit or multiple visits if necessary.
- Questionnaire data was received from 67 schools, covering 7,485 pupils (66.5% of total pupils in the 67 schools).
- The national average FSM % for 11 year old pupils in state funded secondary schools in 2018 was 14.2%[1]. The % for the fitness dataset is 15.4% compared to the whole study sample of 16.8%.

Fitness assessments were completed by 10,697 pupils (girls=57%; eFSM=15.4%) from 74 schools (59 co-educational, 15 single sex). The number of these pupils that also completed the online questionnaire was 7,485 (girls=60%; eFSM=14.3%) from 67 schools (52 co-educational, 15 single sex). Of these, 84.4% took the fitness test and 94.7% answered the questionnaire before the summer vacation. Mean age (SD) at the start of the school year was 12.5 (0.29) years. PA levels during PE were recorded in a total of 88 schools from 111 visits, covering 249 lessons and 9,483 pupils. Pupils were not identified individually, so PA levels could not be examined against fitness or questionnaire scores. 90.1% of visits (85.9% of lessons) were undertaken before the summer vacation.

REFERENCES

1. National Statistics: Schools, pupils and their characteristics: January 2019. Office for National Statistics. https://www.gov.uk/government/statistics/schools-pupils-and-their-characteristics-january-2019/ (accessed 24 Sept 2019).
