# Supplementary material for: Exploring activity levels in physical education lessons in the UK: a cross-sectional examination of activity types and fitness levels
Source: BMJ Open Sport Exerc Med. 2021 Mar 9;7(1):e000924. doi: 10.1136/bmjsem-2020-000924 (PMC7944978; doi:10.1136/bmjsem-2020-000924)
Supplement: Supplementary data [file bmjsem-2020-000924supp002.pdf]

Exploring activity levels in physical education lessons in the UK: A cross-sectional examination of activity types and fitness levels

SUPPLEMENTARY FILE 2: Measures and data cleaning

Physical activity measurements were collected during PE lessons using the wrist-worn AX3 tri-axial accelerometer[1] designed by Open Lab, Newcastle University. The devices were worn on the non-dominant wrist and programmed to sample movement at a frequency of 100Hz. The dynamic range was set at +/- 8g. The raw accelerometry data was downloaded via the manufacturer's software (version AX-GUI-28), after which it was processed into physical activity 'counts' using a one second epoch[2-6] and based on established 'cut-points'[7], via a bespoke LabView programme (National Instruments, Ireland).

The classification of activities is an adaptation of the structure previously set out in the GCSE National Curriculum for Physical Education[8].

- Invasion Games (football, hockey, netball, basketball and rugby)
- Net/wall/racket games (tennis, badminton, table tennis, volleyball)
- Fielding/striking games (cricket, rounders, baseball and softball)
- Athletics (track, field)
- Fitness (circuits, fitness suite, gymnastics, trampolining, dance)
- Adventure (orienteering, other outdoor pursuits)
- Various (unknown or where there is a mix of activities within a lesson)

Data cleaning

Fitness

Baseline dataset (16,017 pupils) -> selected all pupils with a fitness test result (10,697 pupils). Given the large sample size, no outlier analysis was conducted. For fitness model 3, a girls-only dataset was used (6,078 pupils).

PE Enjoyment

Baseline dataset (16,017 pupils) -> selected all pupils with a fitness test result (10,697 pupils) -> selected all pupils with a questionnaire response, excluding outliers that were already tagged in the dataset (7,485 pupils).

Pupil PA during PE lessons

Baseline dataset (16,017 pupils) -> selected all pupils present in PE lessons visited, excluding those who had opted out of the study (9,693 pupils) -> excluded lessons where lesson type was unknown (9,483 pupils). For PE model 4, a location specific dataset (excluding lessons with a mix of location) was used (7,980 pupils).

Exploring activity levels in physical education lessons in the UK: A cross-sectional examination of activity types and fitness levels

REFERENCES

1. Doherty A, Jackson D, Hammerla N, *et al*. Large Scale Population Assessment of Physical Activity Using Wrist Worn Accelerometers: The UK Biobank Study. *PLoS One* 2017;**12**:e0169649.

2. Bailey RC, Olson J, Pepper SL, *et al*. The level and tempo of children's physical activities: an observational study. *Med Sci Sports Exerc.* 1995;**27**:1033-41.

3. Rowlands A, Powell S, Humphries R, *et al*. The Effect of Accelerometer Epoch on Physical Activity Output Measures. *J Exerc Sci Fit.* 2006;**4**:52-58.

4. Stone M, Rowlands AV, Eston R. Relationships between accelerometer-assessed physical activity and health in children: impact of the activity-intensity classification method. *J Sports Sci Med.* 2009;**8**:136-43.

5. Aibar A, Julien C. Physical education: the effect of epoch lengths on children's physical activity in a structured context. *PLoS One* 2015;**10**:e0121238.

6. de Almeida Mendes M, da Silva ICM, Ramires VV, *et al*. Calibration of raw accelerometer data to measure physical activity: A systematic review. *Gait Posture* 2018;**61**:98-110.

7. Phillips LRS, Parfitt G, Rowlands AV. Calibration of the GENEA accelerometer for assessment of physical activity intensity in children. *J Sci Med Sport* 2013;**16:**124-8.

8. GCSE Classification of Sport - Sports Families. S-Cool, the revision website. https://www.s-cool.co.uk/gcse/pe/classification-of-sport/revise-it/sports-families/ (accessed 24 Sept 2019).
