# Supplementary material for: Exploring activity levels in physical education lessons in the UK: a cross-sectional examination of activity types and fitness levels
Source: BMJ Open Sport Exerc Med. 2021 Mar 9;7(1):e000924. doi: 10.1136/bmjsem-2020-000924 (PMC7944978; doi:10.1136/bmjsem-2020-000924)
Supplement: Supplementary data [file bmjsem-2020-000924supp003.pdf]

Exploring activity levels in physical education lessons in the UK: A cross-sectional examination of activity types and fitness levels

SUPPLEMENTARY FILE 3: The Brain Imaging Sub-study

"The Effects of an In-school Physical Activity Intervention on Adolescents' Brain Structure and Function"

Physical activity has shown beneficial effects for cognitive and brain health, suggesting it might provide a highly scalable intervention to improve academic achievement. The sub-study was part of a large-scale randomised controlled trial called 'Fit to Study' (ClinicalTrials.gov ID: NCT03286725). The main 'Fit to Study' trial aimed to test the effect of a school-based physical activity intervention on academic performance (as well as cognition and physical measures) across Year8 pupils in 100 secondary schools. The 'Brain Imaging Sub-study' targeted a sub-sample of participants in the large-scale trial, in order to test pre- to post intervention changes in hippocampal volume, as well as cognitive performance, mental health, brain organisation and cardiorespiratory fitness.

Table: Demographic Data for Schools/Pupils that participated in the Brain imaging sub-study

		**Sub-study Fitness Dataset**		
		**Male**	**Female**	**Total**
School Type (Pupil data)	Co-Ed	30	29	59
	Female	0	1	1
	Male	0	0	0
	Total	30	30	*60
	No	29	30	59
Free School Meals Eligible	Yes	1	0	1
Age, Years (as at 1 Sept. 2017)	Mean (SD)	12.42 (0.28)	12.56 (0.45)	12.49 (0.38)
	Min-Max	12.05-12.95	12.01-14.52	12.01-14.52
Height (cm)	Mean (SD)	153.3 (5.6)	155.3 (7.0)	154.3 (6.4)
	Min-Max	142.0-164.0	140.0-166.0	140.0-166.0
Weight (kg)	Mean (SD)	46.1 (11.4)	46.6 (9.3)	46.4 (10.3)
	Min-Max	31.0-76.0	30.9-70.0	30.9-76.0
BMI (kgm-2)	Mean (SD)	19.65 (4.42)	19.09 (3.06)	19.38 (3.80)
	Min-Max	14.01-32.05	15.77-26.35	14.01-32.05
BMI z-score (entire sample)	Mean (SD)	,04 (1.17)	-,04 (.81)	,00 (1.00)
	Min-Max	-1.41-3.33	-,95-1.83	-1.41-3.33
MAP (mmHg)	Mean (SD)	82.8 (7.1)	84.2 (8.2)	83.5 (7.6)
	Min-Max	71.3-98.3	63.0-102.0	63.0-102.0

Notes
* 60 pupils from across 10 schools

Exploring activity levels in physical education lessons in the UK: A cross-sectional examination of activity types and fitness levels

Table: VO2Max results from cardiopulmonary exercise tests (CPETs)

p = 0.018	**Male (n=30)**	**Female (n=30)**	**Overall (n=60)**
**VO2Max (ml/min/kg)**			
Mean (SD)	39.9 (9.41)	35.0 (6.09)	37.5 (8.25)

Reliability of fitness measure. Concurrent validity testing from CPET

To validate the shuttle run test, a sub-set of pupils (who were participating in a Brain Imaging Sub-study) undertook a cardiopulmonary exercise test to determine their maximal aerobic capacity. This involved a graded test[1, 2] on a cycle ergometer (Lode Excalibur Sport, Groningen, NL). The maximal oxygen uptake (VO2Max) per kilogram was the main dependent variable.

A Bland Altman plot of sub-study pupil fitness (VO2Max) and estimated VO2Max, calculated from the main study fitness data (shuttle run score) using the Leger conversion equation[3] was undertaken, to ascertain if the shuttle run test in schools is a good measure of recording fitness in this age group. Only one data point lied outside the 95% limits, indicating a reasonably accurate agreement between the measures, with a 9.7 (ml/kg/min) bias of overestimation of VO2Max from the shuttle run test.

Table: The results for Bland Altman Plot of the CPET test against the shuttle run test, with confidence intervals

		**SE**	**Lower**	**Upper**
Mean	9.7	0.901	7.832	11.487
SD	5.5			
Lower	-1.084	1.554	-4.236	2.068
Upper	20.403	1.554	17.251	23.555
n	37			
r	0.799		alpha	0.05

REFERENCES

1. Wassenaar TM, Wheatley CM, Beale N, *et al*. Effects of a programme of vigorous physical activity during secondary school physical education on academic performance, fitness, cognition, mental health and the brain of adolescents (Fit to Study): study protocol for a cluster-randomised trial. *Trials* 2019;**20**:189.

2. Godfrey S, Davies C. Estimates of arterial PCO2 and their effect on the calculated values of cardiac output and dead space on exercise. *Clin Sci.* 1970;**39**:529-37.

3. Leger L, Mercier D, Gadoury C, *et al*. The multistage 20 metre shuttle run test for aerobic fitness. *J Sports Sci.* 1988;**6**:93-101.

Exploring activity levels in physical education lessons in the UK: A cross-sectional examination of activity types and fitness levels

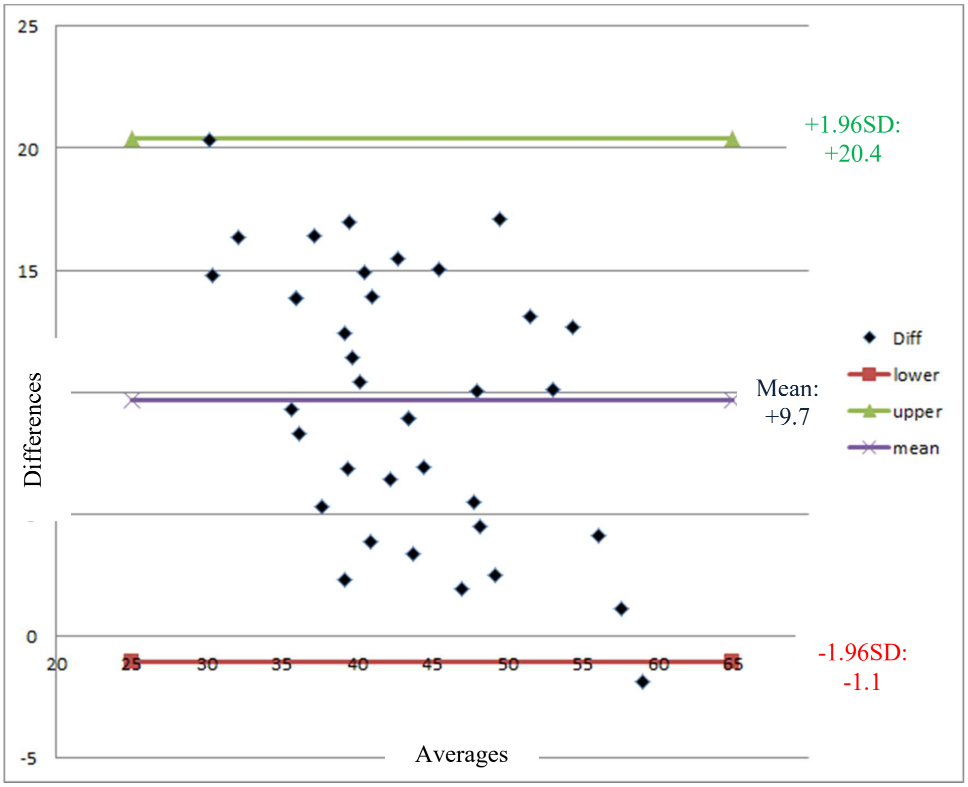

Figure: A Bland Altman Plot of the CPET (x axis) against the shuttle run test results
