# Supplementary material for: Exploring activity levels in physical education lessons in the UK: a cross-sectional examination of activity types and fitness levels
Source: BMJ Open Sport Exerc Med. 2021 Mar 9;7(1):e000924. doi: 10.1136/bmjsem-2020-000924 (PMC7944978; doi:10.1136/bmjsem-2020-000924)
Supplement: Supplementary data [file bmjsem-2020-000924supp004.pdf]

Exploring activity levels in physical education lessons in the UK: A cross-sectional examination of activity types and fitness levels

SUPPLEMENTARY FILE 4: Multi-level model development

Details of model development and specifications, data transformations, and sensitivity analyses for A) Cardiorespiratory fitness (CRF), B) Physical activity (PA) in PE, and C) PE enjoyment.

Statistical analyses were performed in R[1], with lme4[2] using linear mixed effects (LME) analysis. A two-level structure was used for CRF, accounting for clustering at the school level (Level2), while a three-level structure was used for PA in PE classes, accounting for clustering at the school/class level (Level3/Level2). Schools and classes (nested within schools) were treated as having random effects. At the lowest level of the models (Level1), school-level and pupil-level variables, and interactions, were examined as fixed effects.

For all these analyses, data was transformed by the Yeo-Johnson method (VPA) and the orderNorm method (all other PA domains, fitness and PE enjoyment) using the bestNormalize function in R[3]. Pairwise comparisons for the fixed factors, where model estimates indicated significance, were examined as differences of Least Squares (LS) Means adjusted according to Tukey. For all these analyses, a P value inferior to 0.05 was considered statistically significant.

For PA in PE, models were developed for dependent variables SPA, VPA and MVPA, and 'effective' lessons only. Selecting 'effective' lesson data provided the best standardised measure as changing time varied from lesson to lesson, and also presented a 'best case' scenario in terms of PA levels in PE. The three PA domains were chosen because i) VPA more closely predicts CRF than lower intensity activity[4-7], and evidence suggests short bouts of VPA can deliver similar fitness benefits to longer moderate intensity efforts[8, 9], and there are currently no guidelines/recommendations for delivering VPA in PE, ii) MVPA has been, and remains, a focus for most PA research and health guidelines, and iii) reducing sedentary time during the school day is also a focus of research and PA interventions, and SPA has not previously been assessed in secondary school PE.

Development started with null models (intercept only) for the random effect term, and random intercept and fixed slope models were built up, adding group level predictors, and testing model fit improvement at each stage with reference to the Akaike Information Criterion (AIC).

Exploring activity levels in physical education lessons in the UK: A cross-sectional examination of activity types and fitness levels

A) CRF

For CRF, we developed three separate models with fitness (cumulative 'bleep test' laps) as the outcome variable; the inclusion of predictor variables was determined by the research question being addressed. Model°1 was developed to determine how fitness varied i) between boys and girls, ii) with FSM status and iii) to determine the interaction effect of sex with FSM status. Model°2 was developed to explore how fitness varied between school location (based on the Index of Multiple Deprivation (IMD) Tertile[10] for postcode), and the interaction of school type (co-educational or single sex). Model°3 was developed to see if (for girls-only), fitness varied between school type. For all models, school location (based on Defra's Rural-Urban classification[11], school size (based on school year group pupil numbers), the term the fitness test was completed, and pupil age, were included as co-variates. The main predictor variables used in the 'fitness' models were:

- Pupil sex (Male or Female)
- Pupil FSM status (Eligible for FSM: Yes or No)
- School type (Co-educational, Single Sex)
- School postcode IMD tertile (High, Medium, Low - based on the IMD Score)[10]
- The co-variates included in the 'fitness' models were:
- Term of test (Summer 2017 or Autumn 2017)
- Pupil age (as at 1 Sept 2017)
- School location (Urban Major Conurbation, Urban City and Town, Rural Town and Fringe)[11]
- School size (Small (<100pupils in year group), Medium (100-200 pupils), Large (200+ pupils)

The formulae for the three fitness models are presented below:

*Model 1 (Pupil Sex*Pupil FSM status- main effects and interaction)*
Model1<- **lmer(**NormFit$x.t ~ **Sex*FSM** + School.Sex.Type + Tertile + School.Location + SchoolSize + Age + Fitness.1.Term + **(1 + Sex|SchoolID)**, data=Fitness_T1a, REML=FALSE)

*Model 2 (School postcode IMD tertile*School Type- main effects and interaction)*
Model2 <- lmer(NormFit$x.t ~ **Tertile*School.Sex.Type** + Sex + FSM + School.Location + SchoolSize + Age + Fitness.1.Term + **(1 + Sex|SchoolID)**, data=Fitness_T1a, REML=FALSE)

Model 3 (School Type [Girls only dataset])
Model3 <- lmer(NormFit$x.t ~ **School.Sex.Type**+ FSM + Tertile + School.Location + SchoolSize + Age + Fitness.1.Term + **(1|SchoolID)**, data=Fitness_T1a_Girls, REML=FALSE)

Exploring activity levels in physical education lessons in the UK: A cross-sectional examination of activity types and fitness levels

B) PA in PE

We developed four separate models with SPA, VPA, MVPA as the outcome variables; pupils were not identified individually. The inclusion of predictor variables was determined by the research question being addressed. Model°1 was developed to determine how the level of PA in PE lessons varied between school type, the interaction of school FSM status (the proportion of year group pupils eligible for FSM compared to the National average) and lesson type. Model°2 was developed to look at the main effect of activity group on PA levels in PE. Model°3 was developed to investigate the interaction effects of activity by lesson type by school type, however this was reduced to the best interaction according to AIC of activity by lesson type, as the three way interaction did not converge due to school type (single sex) being not represented in mixed classes. Model 4 was developed to explore the interaction of activity and PE Lesson location. The main predictor variables used in the 'PA in PE' models were:

- School type (Co-educational, Single Sex)
- SchoolFSM status (Above or Below National FSM average (all pupils))
- School postcode IMD tertile (High, Medium, Low - based on the IMD Score)[10]
- Lesson type (Boys-only, Girls-only, Mixed)
- Lesson location (Indoors, Outdoors, In/Out)
- Activity group (see Supplementary File 3)

The co-variates included in the 'PA in PE' models were:

- School location (Urban Major Conurbation, Urban City and Town, Rural Town and Fringe)[11]
- School size (Small (<100pupils in year group), Medium (100-200 pupils), Large (200+ pupils)
- Lesson length (<60mins, 60mins, >60mins)

The formulae for the four PE models are presented below:

*Model 1 (School Type + School FSM status*Lesson Type)*
Model1<- lmer(NormFit$x.t~ **SchoolFSM*LessonType + SchoolType** + school.location + Tertile + school.size + LessonLength + ActivityGroup + Location +**(1|SchoolID/LessonID)**, data=PE_Lesson_T1, REML=FALSE)

Model 2 (ActivityGroup)
Model2<- lmer(NormFit$x.t ~ **ActivityGroup**+ Location + LessonType + SchoolType + school.location + SchoolFSMNational + Tertile + school.size + LessonLength+ **(1|SchoolID/LessonID)**, data=PE_Lesson_T1, REML=F)

*Model 3 (ActivityGroup*LessonType)*
Model3<- lmer(NormFit$x.t ~ **ActivityGroup*LessonType**+ SchoolType + school.location + SchoolFSM + Tertile + school.size + LessonLength + Location + **(1|SchoolID/LessonID)**, data=PE_Lesson_T1, REML=F)

*Model 4 (ActivityGroup*Location of PE lesson)*
Model4<- lmer(NormFit$x.t ~ **ActivityGroup*Location**+ LessonType + SchoolType + school.location + SchoolFSMNational + Tertile + school.size + LessonLength+ **(1|SchoolID/LessonID)**, data=PE_Lesson_L3, REML=F)

Exploring activity levels in physical education lessons in the UK: A cross-sectional examination of activity types and fitness levels

C) PE Enjoyment

A multi-level model was used to investigate the effect of PE enjoyment on fitness according to sex.

Table 1: The number of responses, and mean fitness, by PE enjoyment factor

PE Enjoyment			Fitness (cumulative laps) Mean (SD)	Median	[Min, Max]
**One**	Male	(n=71)	27.8 (16.1)	25.0	[2, 98]
	Female	(n=161)	22.7 (12.0)	20.0	[5, 66]
**Two**	Male	(n=48)	26.8 (15.6)	23.5	[3, 73]
	Female	(n=132)	22.6 (9.19)	20.5	[7, 52]
**Three**	Male	(n=68)	31.4 (15.8)	27.0	[8, 77]
	Female	(n=224)	23.5 (10.2)	23.0	[4, 67]
**Four**	Male	(n=202)	30.4 (17.2)	26.5	[0, 125]
	Female	(n=506)	25.5 (11.5)	24.0	[3, 75]
**Five**	Male	(n=189)	31.7 (16.7)	29.0	[7, 103]
	Female	(n=536)	28.4 (13.8)	25.0	[1, 109]
**Six**	Male	(n=640)	38.1 (20.4)	35.0	[0, 120]
	Female	(n=1236)	32.5 (14.9)	30.0	[7, 98]
**Seven**	Male	(n=1772)	48.5 (23.2)	46.0	[0, 137]
	Female	(n=1700)	38.6 (17.4)	36.0	[5, 99]
**Overall**	Male	(n=2990)	42.8 (22.7)	39.0	[0, 137]
	Female	(n=4495)	32.4 (16.1)	29.0	[1, 109]

Table 2: Summary of fitness~PE enjoyment model estimates, CIs and p-values

Model 1 estimate [a]	ß	95% CI
(Intercept)	-2.65***	-3.57 – -1.73
Enjoy PE[b] [Two]	-0.18	-0.49 – 0.13
Enjoy PE [Three]	0.21	-0.07 – 0.49
Enjoy PE [Four]	0.22	-0.01 – 0.45
Enjoy PE [Five]	0.26*	0.03 – 0.49
Enjoy PE [Six]	0.56***	0.36 – 0.77
Enjoy PE [Seven]	1.03***	0.83 – 1.23
Sex[c] [Female]	-0.28*	-0.52 – -0.04
Enjoy PE [Two] * Sex [Female]	0.23	-0.14 – 0.60
Enjoy PE [Three] * Sex [Female]	-0.16	-0.49 – 0.17
Enjoy PE [Four] * Sex [Female]	-0.02	-0.29 – 0.25
Enjoy PE [Five] * Sex [Female]	0.10	-0.18 – 0.37
Enjoy PE [Six] * Sex [Female]	0.01	-0.24 – 0.26
Enjoy PE [Seven] * Sex [Female]	-0.14	-0.39 – 0.10

[a] Fully-adjusted model including age, eFSM, term of test, and school effects; fitness scores are orderNorm transformed
[b] Reference category: Enjoy PE [One]
[c] Reference category: Male
*** p<.001; ** p<.0125; * p <.05

Exploring activity levels in physical education lessons in the UK: A cross-sectional examination of activity types and fitness levels

REFERENCES

1. R Core Team. R: A language and environment for statistical computing. R Foundation for Statistical Computing. 2019.https://www.R-project.org/ (accessed 20 September 2019).

2. Bates D, Mächler M, Bolker B, *et al*. Fitting Linear Mixed-Effects Models Using lme4. *J Stat Softw.* 2015;**67:**1-48

3. Peterson RA, Cavanaugh JE. Ordered quantile normalization: a semiparametric transformation built for the cross-validation era. *J Appl Stat*. 2019;**47**:1-16.

4. Aires L, Silva P, Silva G, *et al*. Intensity of physical activity, cardiorespiratory fitness, and body mass index in youth. *J Phys Act Health* 2010;**7**:54-9.

5. Dencker M, Thorsson O, Karlsson MK, *et al*. Daily physical activity related to aerobic fitness and body fat in an urban sample of children. *Scand J Med Sci Sports* 2008;**18**:728-35.

6. Denton SJ, Trenell MI, Plötz T, *et al*. Cardiorespiratory fitness is associated with hard and light intensity physical activity but not time spent sedentary in 10-14 year old schoolchildren: the HAPPY study. *PLoS One* 2013;**8**:e61073.

7. Gutin B, Yin Z, Humphries MC, *et al*. Relations of moderate and vigorous physical activity to fitness and fatness in adolescents. *Am J Clin Nutr.* 2005;**81**:746-50.

8. Costigan SA, Eather N, Plotnikoff RC, *et al*. High-Intensity Interval Training for Cognitive and Mental Health in Adolescents. *Med Sci Sports Exerc.* 2016;**48**:1985-93.

9. Logan GRM, Nigel H, Scott D, *et al*. A review of adolescent high-intensity interval training. *Sports Med.* 2014;**44**:1071-85.

10. National Statistics: English indices of deprivation 2015. Office for National Statistics. https://www.gov.uk/government/statistics/english-indices-of-deprivation-2015/ (accessed 26 Sept 2019).

11. The 2011 Rural-Urban Classification for Output Areas in England. Office for National Statistics.https://www.ons.gov.uk/methodology/geography/geographicalproducts/ruralurbanclassifications/2011ruralurbanclassification/ (accessed 3 Oct 2019).
