# Supplementary material for: Exploring activity levels in physical education lessons in the UK: a cross-sectional examination of activity types and fitness levels
Source: BMJ Open Sport Exerc Med. 2021 Mar 9;7(1):e000924. doi: 10.1136/bmjsem-2020-000924 (PMC7944978; doi:10.1136/bmjsem-2020-000924)
Supplement: Supplementary data [file bmjsem-2020-000924supp005.pdf]

Exploring activity levels in physical education lessons in the UK: A cross-sectional examination of activity types and fitness levels

SUPPLEMENTARY FILE 5: Demographic data by school variables and lesson length – tables (PA in PE)

Table: (**School-level**) demographic data by school variables, and lesson length

	Lessons Visited (% by column)	School Type: Co-educational	Single Sex	Total
School Size	Small	8 (11.4)	4 (22.2)	12 (13.6)
	Medium	42 (60.0)	10 (55.6)	52 (59.1)
	Large	20 (28.6)	4 (22.2)	24 (27.3)
School FSM	Above Average	40 (51.7)	10 (55.6)	50 (56.8)
	Below Average	30 (42.9)	8 (44.4)	38 (43.2)
IMD Tertile	High	24 (34.3)	7 (38.9)	31 (35.2)
	Medium	17 (24.3)	6 (33.3)	23 (26.1)
	Low	29 (41.4)	5 (27.8)	34 (38.6)
Lesson Length	<60 mins	6 (8.6)	3 (16.7)	9 (10.2)
	60 mins	42 (60.0)	8 (44.4)	50 (56.8)
	>60 mins	22 (31.4)	7 (38.9)	29 (33.0)
	**Total School**	**70**	**18**	**88**

Table: (**Lesson-level**) demographic data by school variables, and lesson length

	Lessons Visited (% by column)	School Type: Co-educational	Single Sex	Total
Activity Group	Invasion games	31 (15.0)	6 (14.0)	37(14.9)
	Net/wall/racket games	14 (6.8)	2 (4.7)	16 (6.4)
	Fielding/striking games	76 (36.9)	11 (25.6)	87 (34.9)
	Athletics	7 (3.4)	5 (11.6)	12 (4.8)
	Fitness	14 (6.8)	2 (4.7)	16 (6.4)
	Adventure/Games	3 (1.5)	1 (2.3)	4 (1.6)
	Various	43 (20.9)	13 (30.2)	56 (22.5)
	Athletics-Field	10 (4.9)	3 (7.0)	13 (5.2)
	Athletics-Track	8 (3.9)	0	8 (3.2)
Lesson Location	Indoors	40 (19.4)	9 (20.9)	49 (19.7)
	Outdoors	149 (72.3)	28 (65.1)	177 (71.1)
	In/Out	17 (8.3)	6 (14.0)	23 (9.2)
Lesson Type	Girls	18 (8.7)	42 (97.7)	60 (24.1)
	Boys	85 (41.3)	1 (2.3)	86 (34.5)
	Mixed	103 (50.0)		103 (41.4)
Lesson Length	<60 mins	18 (8.7)	9 (20.9)	27 (10.8)
	60 mins	119 (57.8)	19 (44.2)	138 (55.4)
	>60 mins	69 (33.5)	15 (34.9)	84 (33.7)
IMD Tertile	High	69 (33.5)	13 (30.2)	82 (32.9)
	Medium	45 (21.8)	17 (39.5)	62 (24.9)
	Low	92 (44.7)	13 (30.2)	105 (42.2)
	**Total Lessons**	**206**	**43**	**249**

Exploring activity levels in physical education lessons in the UK: A cross-sectional examination of activity types and fitness levels

Table: Length of lesson (timetabled versus 'effective' PE time)

**Under 60mins (n=27)**	**60mins (n=138)**	**Above 60mins (n=84)**	**Overall (n=249)**
**Timetabled Lesson Length (minutes)**	**Average of 'effective' PE time (minutes)**	**% of Lesson 'effective' time**	
45	32.8	72.9%	
50	33.6	67.2%	
55	44.0	80.0%	
60	43.4	72.4%	
65	50.5	77.7%	
75	58.0	77.3%	
80	50.4	63.0%	
90	82.2	91.3%	
100	74.9	74.9%	
105	89.7	85.4%	
110	97.1	88.2%	
120	92.9	77.4%	

The above tables show fielding/striking games were by far the most prevalent (34.9% of lessons) followed by invasion games (14.9%) and athletics (13.2%). 6.4% of lessons visited were 'fitness'. The majority of lessons (71.1%) were conducted outdoors. In co-educational schools, 50.0% of lessons visited were 'mixed' sex composition, compared to 8.7% for girls-only lessons. The balance of lesson visits to schools located across the postcode IMD tertile ratings were: high 35.2%, medium 26.1% and low 38.6%. 86.4% of schools contained more than 100 pupils in the year group, and 89.8% of timetabled lessons were 60 minutes and above.
