# Supplementary material for: Exploring activity levels in physical education lessons in the UK: a cross-sectional examination of activity types and fitness levels
Source: BMJ Open Sport Exerc Med. 2021 Mar 9;7(1):e000924. doi: 10.1136/bmjsem-2020-000924 (PMC7944978; doi:10.1136/bmjsem-2020-000924)
Supplement: Supplementary data [file bmjsem-2020-000924supp006.pdf]

Exploring activity levels in physical education lessons in the UK: A cross-sectional examination of activity types and fitness levels

SUPPLEMENTARY FILE 6: Demographic data by school and lesson variables – tables (PA in PE)

Tables: (**Lesson-level**) demographic data by school and lesson variables

No. of Lessons	School Type		Tertile			Lesson Location			
ActivityGroup	Co-Ed (n=206)	Single Sex (n=43)	High (n=82)	Medium (n=62)	Low (n=105)	Indoors (n=49)	Outdoors (n=177)	In/Out (n=23)	Overall (n=249)
Invasion Games	31 (15.0%)	6 (14.0%)	13 (15.9%)	15 (24.2%)	9 (8.6%)	9 (18.4%)	28 (15.8%)	0 (0%)	37 (14.9%)
Net/wall/racket games	14 (6.8%)	2 (4.7%)	4 (4.9%)	1 (1.6%)	11 (10.5%)	6 (12.2%)	10 (5.6%)	0 (0%)	16 (6.4%)
Fielding/striking games	76 (36.9%)	11 (25.6%)	25 (30.5%)	25 (40.3%)	37 (35.2%)	9 (18.4%)	76 (42.9%)	2 (8.7%)	87 (34.9%)
Athletics	7 (3.4%)	5 (11.6%)	2 (2.4%)	5 (8.1%)	5 (4.8%)	0 (0%)	12 (6.8%)	0 (0%)	12 (4.8%)
Fitness	14 (6.8%)	2 (4.7%)	4 (4.9%)	0 (0%)	12 (11.4%)	15 (30.6%)	1 (0.6%)	0 (0%)	16 (6.4%)
Adventure/Games	3 (1.5%)	1 (2.3%)	1 (1.2%)	0 (0%)	3 (2.9%)	2 (4.1%)	2 (1.1%)	0 (0%)	4 (1.6%)
Various	43 (20.9%)	13 (30.2%)	19 (23.2%)	14 (22.6%)	23 (21.9%)	4 (8.2%)	31 (17.5%)	21 (91.3%)	56 (22.5%)
Athletics-Field	10 (4.9%)	3 (7.0%)	10 (12.2%)	1 (1.6%)	2 (1.9%)	4 (8.2%)	9 (5.1%)	0 (0%)	13 (5.2%)
Athletics-Track	8 (3.9%)	0 (0%)	4 (4.9%)	1 (1.6%)	3 (2.9%)	0 (0%)	8 (4.5%)	0 (0%)	8 (3.2%)

No. of Lessons	Lesson Type			
ActivityGroup	Girls (n=60)	Boys (n=86)	Mixed (n=103)	Overall (n=249)
Invasion Games	13 (21.7%)	19 (22.1%)	5 (4.9%)	37 (14.9%)
Net/wall/racket games	3 (5.0%)	8 (9.3%)	5 (4.9%)	16 (6.4%)
Fielding/striking games	17 (28.3%)	37 (43.0%)	33 (32.0%)	87 (34.9%)
Athletics	5 (8.3%)	4 (4.7%)	3 (2.9%)	12 (4.8%)
Fitness	4 (6.7%)	8 (9.3%)	4 (3.9%)	16 (6.4%)
Adventure/Games	1 (1.7%)	2 (2.3%)	1 (1.0%)	4 (1.6%)
Various	14 (23.3%)	1 (1.2%)	41 (39.8%)	56 (22.5%)
Athletics-Field	3 (5.0%)	5 (5.8%)	5 (4.9%)	13 (5.2%)
Athletics-Track	0 (0%)	2 (2.3%)	6 (5.8%)	8 (3.2%)

Exploring activity levels in physical education lessons in the UK: A cross-sectional examination of activity types and fitness levels

Tables: (**Pupil-level**) demographic data by school and lesson variables

**No. of Pupils**	**School Type**		**Tertile**			**Lesson Location**			
**ActivityGroup**	**Co-Ed (n=8059)**	**Single Sex (n=1424)**	**High (n=2769)**	**Medium (n=2150)**	**Low (n=4564)**	**Indoors (n=1565)**	**Outdoors (n=6415)**	**In/Out (n=1503)**	**Overall (n=9483)**
Invasion Games	920 (11.4%)	165 (11.6%)	351 (12.7%)	400 (18.6%)	334 (7.3%)	322 (20.6%)	763 (11.9%)	0 (0%)	1085 (11.4%)
Net/wall/racket games	347 (4.3%)	47 (3.3%)	108 (3.9%)	27 (1.3%)	259 (5.7%)	145 (9.3%)	249 (3.9%)	0 (0%)	394 (4.2%)
Fielding/striking games	2539 (31.5%)	373 (26.2%)	738 (26.7%)	696 (32.4%)	1478 (32.4%)	348 (22.2%)	2453 (38.2%)	111 (7.4%)	2912 (30.7%)
Athletics	219 (2.7%)	110 (7.7%)	73 (2.6%)	110 (5.1%)	146 (3.2%)	0 (0%)	329 (5.1%)	0 (0%)	329 (3.5%)
Fitness	458 (5.7%)	43 (3.0%)	106 (3.8%)	0 (0%)	395 (8.7%)	468 (29.9%)	33 (0.5%)	0 (0%)	501 (5.3%)
Adventure/Games	96 (1.2%)	16 (1.1%)	16 (0.6%)	0 (0%)	96 (2.1%)	47 (3.0%)	65 (1.0%)	0 (0%)	112 (1.2%)
Various	2977 (36.9%)	586 (41.2%)	1037 (37.5%)	881 (41.0%)	1645 (36.0%)	160 (10.2%)	2011 (31.3%)	1392 (92.6%)	3563 (37.6%)
Athletics-Field	228 (2.8%)	84 (5.9%)	220 (7.9%)	20 (0.9%)	72 (1.6%)	75 (4.8%)	237 (3.7%)	0 (0%)	312 (3.3%)
Athletics-Track	275 (3.4%)	0 (0%)	120 (4.3%)	16 (0.7%)	139 (3.0%)	0 (0%)	275 (4.3%)	0 (0%)	275 (2.9%)

**No. of Pupils**	**Lesson Type**			
**ActivityGroup**	**Girls (n=1961)**	**Boys (n=2446)**	**Mixed (n=5076)**	**Overall (n=9483)**
Invasion Games	328 (16.7%)	621 (25.4%)	136 (2.7%)	1085 (11.4%)
Net/wall/racket games	73 (3.7%)	191 (7.8%)	130 (2.6%)	394 (4.2%)
Fielding/striking games	601 (30.6%)	1058 (43.3%)	1253 (24.7%)	2912 (30.7%)
Athletics	110 (5.6%)	105 (4.3%)	114 (2.2%)	329 (3.5%)
Fitness	88 (4.5%)	243 (9.9%)	170 (3.3%)	501 (5.3%)
Adventure/Games	16 (0.8%)	55 (2.2%)	41 (0.8%)	112 (1.2%)
Various	661 (33.7%)	20 (0.8%)	2882 (56.8%)	3563 (37.6%)
Athletics-Field	84 (4.3%)	93 (3.8%)	135 (2.7%)	312 (3.3%)
Athletics-Track	0 (0%)	60 (2.5%)	215 (4.2%)	275 (2.9%)
