# Supplementary material for: Exploring activity levels in physical education lessons in the UK: a cross-sectional examination of activity types and fitness levels
Source: BMJ Open Sport Exerc Med. 2021 Mar 9;7(1):e000924. doi: 10.1136/bmjsem-2020-000924 (PMC7944978; doi:10.1136/bmjsem-2020-000924)
Supplement: Supplementary data [file bmjsem-2020-000924supp007.pdf]

Exploring activity levels in physical education lessons in the UK: A cross-sectional examination of activity types and fitness levels

SUPPLEMENTARY FILE 7: Fitness model estimates

Table: Summary of fitness model estimates, CIs and p-values

**Model 1 estimate [a]**	**ß**	**95% CI**
(Intercept)	-2.37***	-3.18 to -1.56
Female[b]	-0.48***	-0.56 to -0.39
eFSM[c]	-0.22***	-0.29 to -0.15
Female*eFSM	-0.01	-0.11 to 0.09

[a] Fully-adjusted model including age, term of test, and school effects; fitness scores are orderNorm transformed
[b] Reference category: male
[c] Reference category: not eligible for FSM
*** p<.001; ** p<.0125; * p <.05

**Model 2 estimate [a]**	**ß**	**95% CI**
(Intercept)	-2.34***	-3.16 to -1.52
Tertile-Low[b]	-0.40**	0.16 to 0.63
School Type (ST)[c]	-0.11	-0.48 to 0.26
Tertile-Medium*ST	0.27	-0.31 to 0.84
Tertile-Low*ST	-0.03	-0.55 to -0.49

[a] Fully-adjusted model including sex, FSM, age, term of test, and school effects; fitness scores are orderNorm transformed
[b] Reference category: High
[c] Reference category: Co-educational
*** p<.001; ** p<.0125; * p <.05

**Model 3 estimate [a]**	**ß**	**95% CI**
(Intercept)	-1.95***	-3.03 to -0.88
School Type[b]	-0.02	-0.30 to 0.26

[a] Fully-adjusted model including FSM, age, term of test, and school effects; fitness scores are orderNorm transformed
[b] Reference category: Co-educational
*** p<.001; ** p<.0125; * p <.05

Notes

*Fitness Model 1 (Pupil Sex*Pupil FSM status - main effects and interaction)* to determine how fitness varied i) between boys and girls, ii) with FSM status and iii) to determine the interaction effect of sex with FSM status on fitness.

*Fitness Model 2 (School postcode Index of Multiple Deprivation (IMD) Tertile*School Type - main effects and interaction) to* explore how fitness varied between school location (based on the IMD tertile), and the interaction of school type (co-educational or single sex).

Fitness Model 3 (School Type [Girls only]) was developed to see if, for girls-only, fitness varied between school type.
