# Supplementary material for: Exploring activity levels in physical education lessons in the UK: a cross-sectional examination of activity types and fitness levels
Source: BMJ Open Sport Exerc Med. 2021 Mar 9;7(1):e000924. doi: 10.1136/bmjsem-2020-000924 (PMC7944978; doi:10.1136/bmjsem-2020-000924)
Supplement: Supplementary data [file bmjsem-2020-000924supp008.pdf]

Exploring activity levels in physical education lessons in the UK: A cross-sectional examination of activity types and fitness levels

## SUPPLEMENTARY FILE 8: Pupil average time (%) for school/lesson variables - tables

**Tables:** (**Pupil-level**) pupil average time (%) in PE lessons split by PA domain, for school/lesson variables

| | School Type | | Tertile | | |
|---|---|---|---|---|---|
| **% of 1hr Lesson (SD)** | **Co-Ed (n=8059)** | **Single Sex (n=1424)** | **High (n=2769)** | **Medium (n=2150)** | **Low (n=4564)** |
| **PA Domains** | | | | | |
| SPA | 44.0 (13.3) | 45.0 (13.0) | 44.5 (14.3) | 43.0 (12.2) | 44.5 (13.1) |
| LPA | 31.9 (7.77) | 32.5 (7.13) | 32.2 (7.82) | 32.9 (7.23) | 31.4 (7.76) |
| MPA | 16.7 (6.46) | 16.5 (6.54) | 16.6 (6.72) | 17.0 (6.07) | 16.6 (6.49) |
| VPA | 7.34 (4.55) | 6.01 (3.72) | 6.68 (4.56) | 7.12 (4.09) | 7.44 (4.54) |
| MVPA | 24.1 (9.45) | 22.5 (9.19) | 23.3 (10.1) | 24.1 (8.83) | 24.1 (9.24) |

| | Lesson Type | | | |
|---|---|---|---|---|
| **% of 1hr Lesson (SD)** | **Girls (n=1961)** | **Boys (n=2446)** | **Mixed (n=5076)** | **Overall (n=9483)** |
| SPA | 44.7 (12.7) | 42.3 (13.2) | 44.9 (13.4) | 44.2 (13.3) |
| LPA | 32.6 (7.09) | 33.0 (7.71) | 31.2 (7.81) | 32.0 (7.68) |
| MPA | 16.3 (6.36) | 16.9 (6.32) | 16.8 (6.57) | 16.7 (6.47) |
| VPA | 6.48 (3.97) | 7.78 (4.50) | 7.09 (4.58) | 7.14 (4.46) |
| MVPA | 22.7 (9.11) | 24.7 (9.50) | 23.9 (9.47) | 23.8 (9.42) |

Exploring activity levels in physical education lessons in the UK: A cross-sectional examination of activity types and fitness levels

**Tables:** (**Lesson-level**) pupil average time (%) in PE lessons split by PA domain, for school/lesson variables

| | **School Type** | | **Tertile** | | |
|---|---|---|---|---|---|
| **% of 1hr Lesson (SD)** | **Co-Ed (n=206)** | **Single Sex (n=43)** | **High (n=82)** | **Medium (n=62)** | **Low (n=105)** |
| **PA Domains** | | | | | |
| SPA | 43.8 (8.96) | 46.6 (8.46) | 45.5 (10.3) | 43.2 (8.34) | 43.9 (8.02) |
| LPA | 32.3 (4.56) | 31.8 (3.74) | 31.7 (4.65) | 33.1 (4.20) | 32.0 (4.34) |
| MPA | 16.9 (4.25) | 16.1 (4.41) | 16.3 (4.47) | 17.2 (4.11) | 16.8 (4.22) |
| VPA | 7.31 (3.05) | 5.56 (2.11) | 6.47 (2.94) | 6.99 (2.87) | 7.44 (3.05) |
| MVPA | 24.2 (6.30) | 21.6 (5.75) | 22.7 (6.76) | 24.2 (6.28) | 24.2 (5.83) |

| | **Lesson Type** | | | |
|---|---|---|---|---|
| **% of 1hr Lesson (SD)** | **Girls (n=60)** | **Boys (n=86)** | **Mixed (n=103)** | **Overall (n=249)** |
| SPA | 45.3 (8.61) | 42.8 (9.31) | 44.8 (8.70) | 44.3 (8.93) |
| LPA | 32.3 (3.63) | 32.9 (4.78) | 31.5 (4.47) | 32.2 (4.43) |
| MPA | 16.2 (4.37) | 16.9 (4.47) | 16.9 (4.07) | 16.7 (4.28) |
| VPA | 6.20 (2.61) | 7.70 (3.19) | 6.89 (2.91) | 7.00 (2.98) |
| MVPA | 22.4 (6.11) | 24.6 (6.68) | 23.8 (5.93) | 23.7 (6.27) |
