# Supplementary material for: Exploring activity levels in physical education lessons in the UK: a cross-sectional examination of activity types and fitness levels
Source: BMJ Open Sport Exerc Med. 2021 Mar 9;7(1):e000924. doi: 10.1136/bmjsem-2020-000924 (PMC7944978; doi:10.1136/bmjsem-2020-000924)
Supplement: Supplementary data [file bmjsem-2020-000924supp009.pdf]

SUPPLEMENTARY FILE 9: Pupil average time by activity group - tables and violin plots

Table. Pupil average time (mins/hr) of PA domains in PE lessons split by activity group

Time [mins/hr] (SD)	Invasion Games (n=1085)	Net/wall/racket games (n=394)	Fielding/striking games (n=2912)	Athletics (n=329)	Fitness (n=501)	Adventure/ Games (n=112)	Various (n=3563)	Athletics-Field (n=312)	Athletics-Track (n=275)	Overall (n=9483)
**SPA**	22.2 (7.64)	23.7 (8.30)	27.5 (7.15)	28.2 (5.74)	25.3 (6.85)	30.8 (12.2)	26.4 (8.00)	34.1 (8.72)	28.2 (6.75)	26.5 (7.97)
**LPA**	20.8 (4.62)	20.6 (4.42)	19.2 (4.01)	18.7 (4.08)	18.0 (4.83)	17.8 (6.88)	19.2 (4.72)	16.1 (5.17)	18.5 (4.77)	19.2 (4.61)
**MPA**	11.5 (3.79)	12.2 (4.67)	9.46 (3.50)	8.81 (2.62)	10.6 (4.29)	7.47 (3.66)	10.3 (3.89)	7.10 (3.31)	8.54 (3.15)	10.0 (3.88)
**VPA**	5.50 (3.29)	3.48 (2.23)	3.90 (2.16)	4.37 (2.11)	6.11 (3.77)	4.01 (3.06)	4.17 (2.49)	2.70 (1.91)	4.74 (3.29)	4.29 (2.68)
**MVPA**	17.0 (6.22)	15.7 (6.38)	13.4 (4.97)	13.2 (3.64)	16.7 (5.81)	11.5 (6.41)	14.4 (5.58)	9.80 (4.82)	13.3 (5.02)	14.3 (5.65)

Table. Pupil average % of PA domains in PE lessons split by activity group

% of Lesson (SD)	Invasion Games (n=1085)	Net/wall/racket games (n=394)	Fielding/striking games (n=2912)	Athletics (n=329)	Fitness (n=501)	Adventure/ Games (n=112)	Various (n=3563)	Athletics-Field (n=312)	Athletics-Track (n=275)	Overall (n=9483)
**SPA**	37.0 (12.7)	39.5 (13.8)	45.8 (11.9)	46.9 (9.56)	42.1 (11.4)	51.3 (20.4)	44.0 (13.3)	56.8 (14.5)	47.0 (11.3)	44.2 (13.3)
**LPA**	34.6 (7.69)	34.3 (7.37)	32.0 (6.68)	31.1 (6.80)	30.0 (8.05)	29.6 (11.5)	31.9 (7.87)	26.8 (8.62)	30.8 (7.96)	32.0 (7.68)
**MPA**	19.2 (6.31)	20.4 (7.79)	15.8 (5.83)	14.7 (4.37)	17.7 (7.15)	12.4 (6.10)	17.1 (6.48)	11.8 (5.52)	14.2 (5.25)	16.7 (6.47)
**VPA**	9.16 (5.49)	5.81 (3.72)	6.50 (3.60)	7.29 (3.51)	10.2 (6.29)	6.68 (5.10)	6.95 (4.15)	4.50 (3.19)	7.90 (5.48)	7.14 (4.46)
**MVPA**	28.4 (10.4)	26.2 (10.6)	22.3 (8.29)	22.0 (6.06)	27.9 (9.69)	19.1 (10.7)	24.0 (9.31)	16.3 (8.03)	22.1 (8.36)	23.8 (9.42)

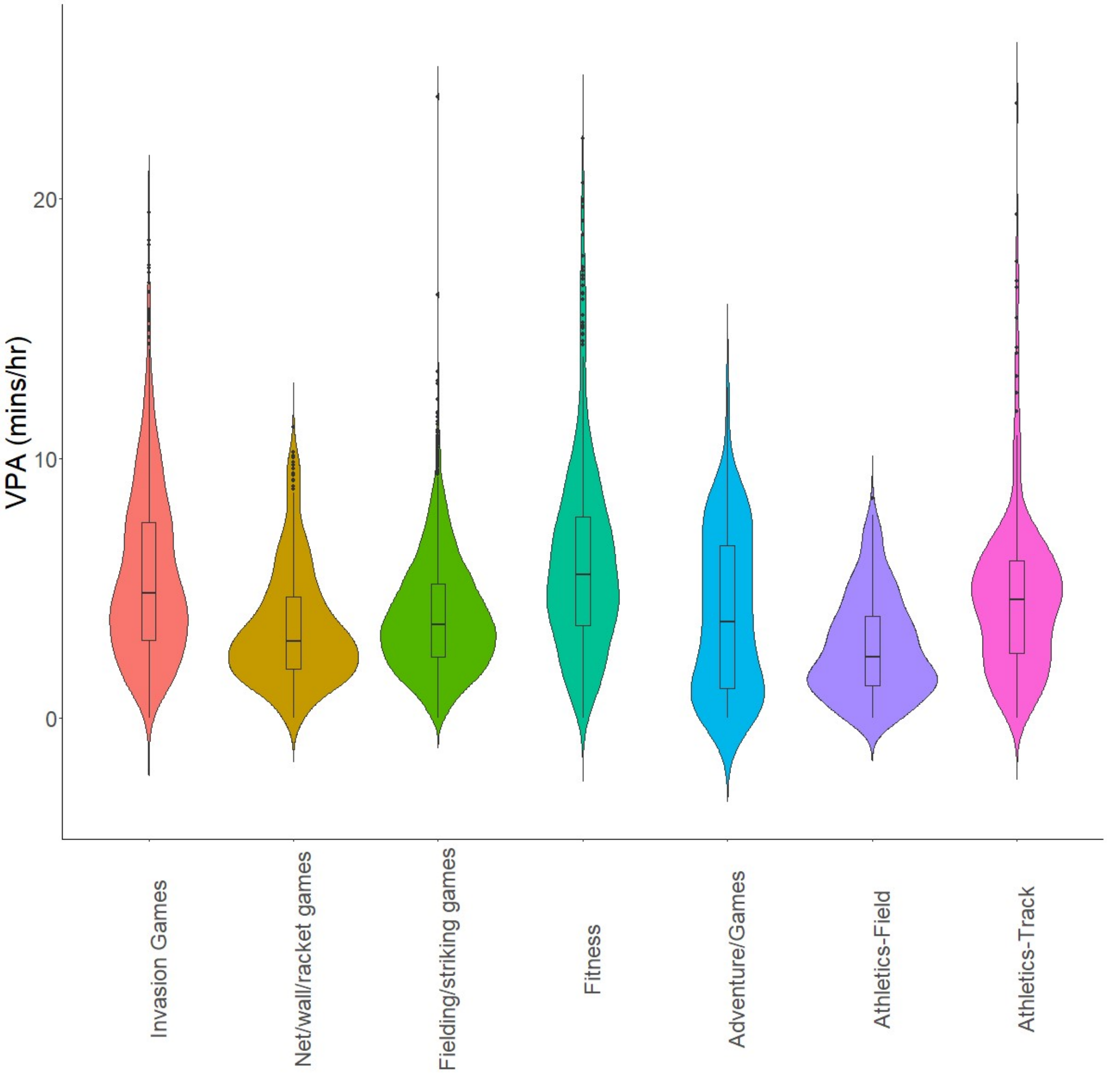

Figure: Violin plot of pupil average time (mins/hr) in PE lessons split by activity group for VPA

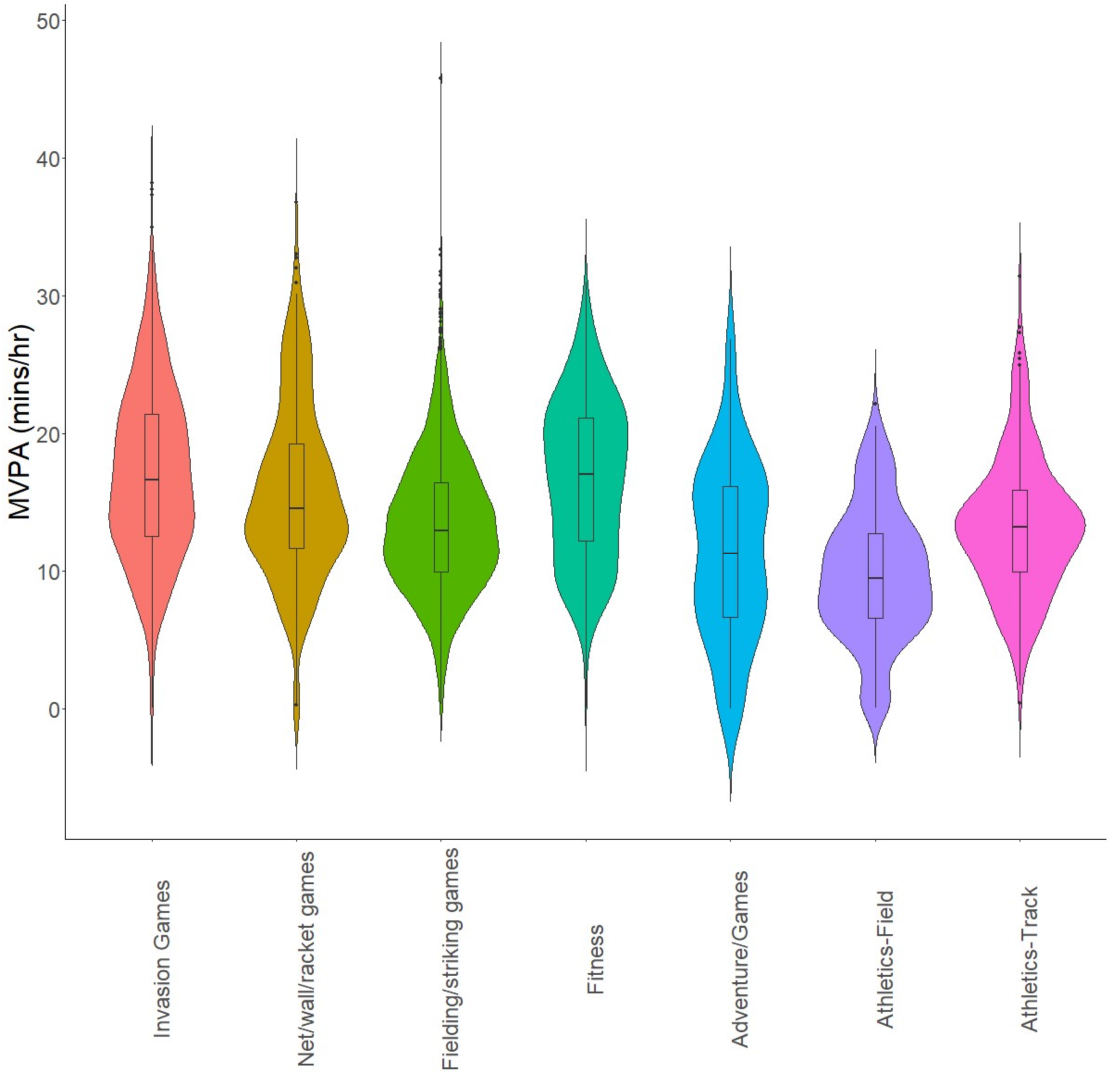

Figure: Violin plot of pupil average time (mins/hr) in PE lessons split by activity group for MVPA

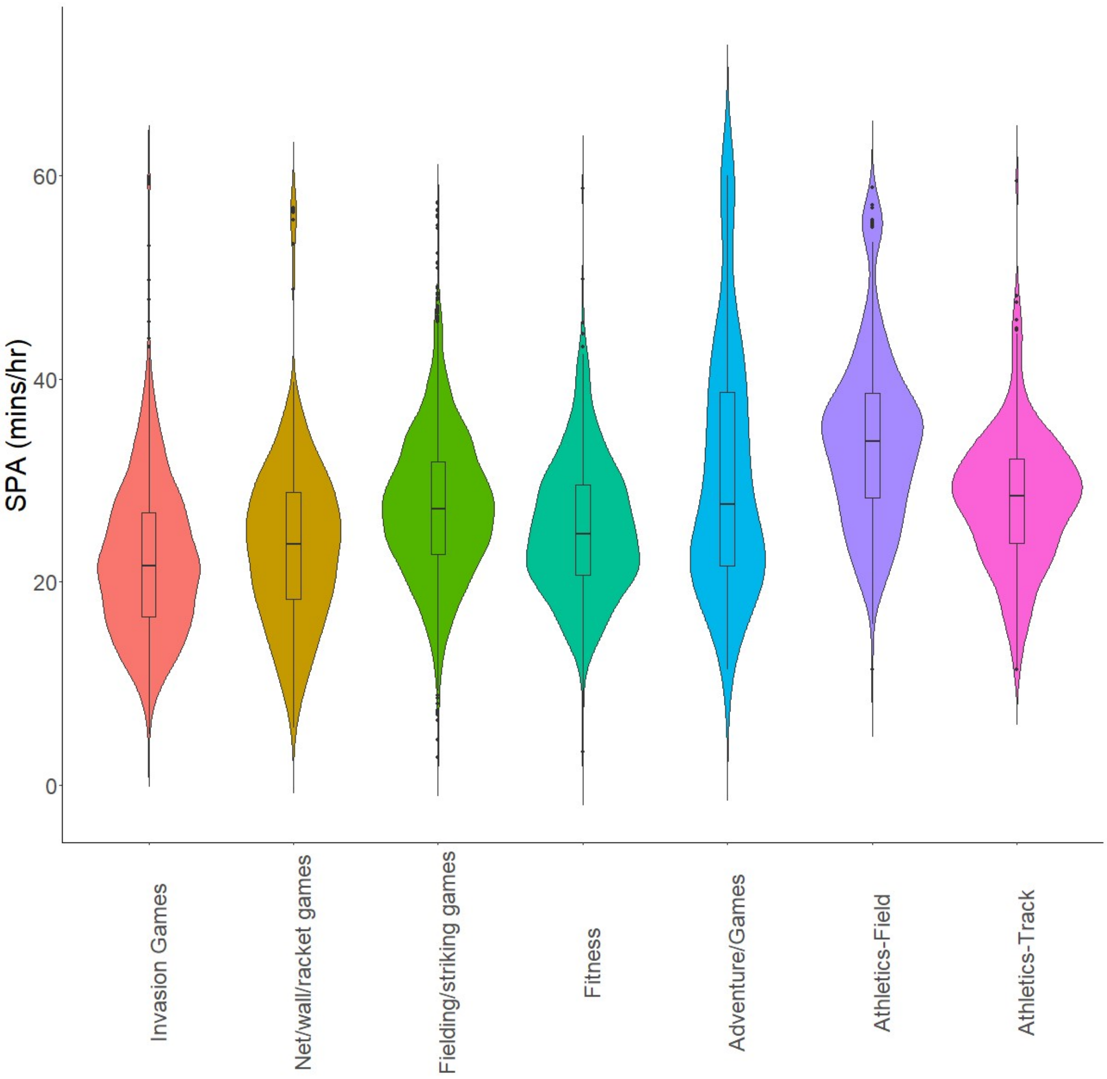

Figure: Violin plot of pupil average time (mins/hr) in PE lessons split by activity group for SPA
