# Supplementary material for: Exploring activity levels in physical education lessons in the UK: a cross-sectional examination of activity types and fitness levels
Source: BMJ Open Sport Exerc Med. 2021 Mar 9;7(1):e000924. doi: 10.1136/bmjsem-2020-000924 (PMC7944978; doi:10.1136/bmjsem-2020-000924)
Supplement: Supplementary data [file bmjsem-2020-000924supp010.pdf]

Exploring activity levels in physical education lessons in the UK: A cross-sectional examination of activity types and fitness levels

SUPPLEMENTARY FILE 10: PA in PE model estimates

Tables: Summary of PA in PE model estimates, CIs and p-values

	VPA		MVPA		SPA	
**Table 1 | Model 1 estimate** [a]	**ß**	**95% CI**	**ß**	**95% CI**	**ß**	**95% CI**
(Intercept)	0.00	-0.61 to 0.62	0.04	-0.51 to 0.59	0.06	-0.47 to 0.59
School Type[b]	-0.18	-0.62 to 0.26	-0.02	-0.42 to 0.37	0.05	-0.33 to 0.43
School FSM[c]	-0.02	-0.42 to 0.39	0.02	-0.35 to 0.38	-0.02	-0.37 to 0.33
Lesson Type-Boys[d]	0.23	-0.24 to 0.71	0.25	-0.17 to 0.68	-0.21	-0.63 to 0.20
Lesson Type-Mixed[d]	-0.08	-0.54 to 0.39	0.10	-0.32 to 0.52	-0.01	-0.42 to 0.40
School FSM*Lesson Type-Boys	-0.15	-0.68 to 0.39	-0.15	-0.62 to 0.33	0.18	-0.28 to 0.64
School FSM*Lesson Type-Mixed	0.15	-0.37 to 0.67	0.04	-0.43 to 0.51	-0.00	-0.46 to 0.45

[a] Fully-adjusted model including lesson length, lesson location, activity and school effects; SPA/MVPA are orderNorm transformed; VPA are Yeo-Johnson transformed
[b] Reference category: Co-educational; [c] Reference category: Above average; [d] Reference category: Girls-only
*** p<.001; ** p<.0125; * p <.05

	VPA		MVPA		SPA	
**Table 2 | Model 2 estimate** [a]	**ß**	**95% CI**	**ß**	**95% CI**	**ß**	**95% CI**
(Intercept)	0.04	-0.56 to 0.64	0.08	-0.45 to 0.62	0.01	-0.51 to 0.53
Net/wall/racket games[b]	-0.38*	-0.68 to -0.07	-0.16	-0.46 to 0.13	0.10	-0.19 to 0.39
Fielding/striking games[b]	-0.49***	-0.74 to -0.24	-0.55***	-0.79 to -0.32	0.54***	0.31 to 0.77
Athletics	-0.09	-0.47 to 0.29	-0.45*	-0.81 to -0.08	0.58**	0.22 to 0.94
Fitness[b]	0.29	-0.06 to 0.63	0.22	-0.11 to 0.55	-0.18	-0.51 to 0.14
Adventure/Games[b]	-0.50	-1.06 to 0.06	-0.81**	-1.35 to -0.27	0.70**	0.17 to 1.24
Various	-0.40**	-0.71 to -0.09	-0.47**	-0.76 to -0.18	0.55***	0.26 to 0.83
Athletics-Field[b]	-0.81***	-1.23 to -0.39	-1.17***	-1.56 to -0.78	1.34***	0.95 to 1.72
Athletics-Track[b]	0.38*	0.01 to 0.74	0.02	-0.33 to 0.37	0.24	-0.10 to 0.58

[a] Fully-adjusted model including lesson length, lesson location, activity and school effects; SPA/MVPA are orderNorm transformed; VPA are Yeo-Johnson transformed.
[b] Reference category: Invasion games
*** p<.001; ** p<.0125; * p <.05
Significant differences observed for activity group; post-hoc analysis conducted – see Table 4.

Exploring activity levels in physical education lessons in the UK: A cross-sectional examination of activity types and fitness levels

**Table 3 | Model 3 estimate** [a]	VPA ß	VPA 95% CI	MVPA ß	MVPA 95% CI	SPA ß	SPA 95% CI
(Intercept)	0.17	-0.40 to 0.74	0.19	-0.16 to 1.09	0.00	-0.54 to 0.54
Net/wall/racket games[b]	-0.10	-0.74 to 0.54	0.46	-1.04 to -0.22	-0.39	-1.02 to 0.25
Fielding/striking games[b]	-0.29	-0.72 to 0.13	-0.63**	-0.80 to 0.42	0.61**	0.19 to 1.03
Athletics[b]	0.15	-0.48 to 0.78	-0.19	-1.12 to -0.01	0.30	-0.32 to 0.92
Fitness[b]	-0.57*	-1.13 to -0.00	-0.57*	-1.77 to 0.30	0.10	-0.46 to 0.66
Adventure/Games[b]	-0.49	-1.55 to 0.56	-0.73	-0.74 to 0.14	0.46	-0.58 to 1.50
Various[b]	-0.14	-0.60 to 0.32	-0.30	-1.46 to -0.19	0.32	-0.13 to 0.77
Athletics-Field[b]	-0.30	-0.96 to 0.35	-0.82**	-0.44 to 0.53	1.07***	0.42 to 1.71
Athletics-Track[b]	0.34	-0.15 to 0.83	0.04	-0.29 to 0.62	0.13	-0.36 to 0.61
Lesson Type-Boys[c]	0.25	-0.24 to 0.73	0.16	-0.44 to 0.69	-0.20	-0.66 to 0.27
Lesson Type-Mixed[c]	0.05	-0.53 to 0.63	0.13	-0.52 to 0.24	0.03	-0.54 to 0.60
School Type[d]	-0.37	-0.78 to 0.04	-0.14	-1.52 to 0.02	0.14	-0.25 to 0.53
ActivityGroupNet/wall/racket games:LessonTypeBoys[e]	-0.48	-1.27 to 0.32	-0.75	-0.32 to 0.70	0.54	-0.24 to 1.32
ActivityGroupFielding/striking games:LessonTypeBoys[e]	-0.05	-0.58 to 0.49	0.19	-1.20 to 0.42	-0.06	-0.58 to 0.46
ActivityGroupAthletics:LessonTypeBoys[e]	-0.36	-1.20 to 0.48	-0.39	0.27 to 1.64	0.40	-0.43 to 1.22
ActivityGroupFitness:LessonTypeBoys[e]	0.70	-0.00 to 1.40	0.96**	-1.58 to 0.93	-0.46	-1.15 to 0.23
ActivityGroupAdventure/Games:LessonTypeBoys[e]	-0.41	-1.70 to 0.88	-0.33	-2.12 to 0.07	0.54	-0.73 to 1.82
ActivityGroupVarious:LessonTypeBoys[e]	-0.99	-2.12 to 0.14	-1.02	-1.53 to 0.34	1.28*	0.17 to 2.39
ActivityGroupAthletics-Field:LessonTypeBoys[e]	-1.03*	-2.02 to -0.05	-0.60	-1.40 to 0.34	0.59	-0.35 to 1.54
ActivityGroupAthletics-Track:LessonTypeBoys[e]	-0.68	-1.57 to 0.21	-0.53	-1.55 to 0.06	0.32	-0.56 to 1.19
ActivityGroupNet/wall/racket games:LessonTypeMixed[e]	-0.33	-1.14 to 0.49	-0.74	-0.57 to 0.64	0.53	-0.28 to 1.33
ActivityGroupFielding/striking games:LessonTypeMixed[e]	-0.36	-0.98 to 0.26	0.04	-1.18 to 0.64	-0.19	-0.79 to 0.42
ActivityGroupAthletics:LessonTypeMixed[e]	-0.10	-1.04 to 0.84	-0.27	0.26 to 1.87	0.32	-0.60 to 1.24
ActivityGroupFitness:LessonTypeMixed[e]	1.69***	0.86 to 2.51	1.06**	-1.18 to 1.72	-0.19	-1.00 to 0.63
ActivityGroupAdventure/Games:LessonTypeMixed[e]	0.49	-0.99 to 1.98	0.27	-0.81 to 0.41	-0.25	-1.71 to 1.22
ActivityGroupVarious:LessonTypeMixed[e]	-0.25	-0.88 to 0.38	-0.20	-1.40 to 0.42	0.19	-0.42 to 0.81
ActivityGroupAthletics-Field:LessonTypeMixed[e]	-0.64	-1.58 to 0.29	-0.49	-0.16 to 1.09	0.19	-0.73 to 1.11

[a] Fully-adjusted model including lesson length, lesson location, and school effects; SPA/MVPA are orderNorm transformed; VPA are Yeo-Johnson transformed.
[b] Reference category: Invasion games [c] Reference category: Girls-only [d] Reference category: Co-educational
[e] Reference category: ActivityGroupInvasion games:LessonTypeGirls
*** p<.001; ** p<.0125; * p <.05
Significant differences observed for ActivityGroup*LessonType; post-hoc analysis conducted
