# Supplementary material for: Exploring activity levels in physical education lessons in the UK: a cross-sectional examination of activity types and fitness levels
Source: BMJ Open Sport Exerc Med. 2021 Mar 9;7(1):e000924. doi: 10.1136/bmjsem-2020-000924 (PMC7944978; doi:10.1136/bmjsem-2020-000924)
Supplement: Supplementary data [file bmjsem-2020-000924supp011.pdf]

Exploring activity levels in physical education lessons in the UK: A cross-sectional examination of activity types and fitness levels

SUPPLEMENTARY FILE 11: LSMeans tables

Tables: summary of the fixed effect interactions (LS Means) for Models 2, 3 and 4, for VPA, MVPA & SPA

Table 1 | Model 2 | Activity Group | VPA | lsmeans

Activity Group	lsmean	SE	df	lower.CL	upper.CL
Invasion games	0.1244	0.162	219	-0.1945	0.44336
Net/wall/racket games	-0.2481	0.169	311	-0.5807	0.08456
Fielding/striking games	-0.3645	0.135	169	-0.631	-0.09806
Athletics	0.0354	0.201	276	-0.3598	0.43056
Fitness	0.4115	0.203	273	0.0126	0.8103
Adventure/Games	-0.3718	0.3	264	-0.9616	0.21798
Various	-0.2662	0.137	163	-0.5374	0.00492
Athletics-Field	-0.679	0.225	225	-1.1214	-0.23652
Athletics-Track	0.5036	0.197	368	0.1169	0.89035

Table 2 | Model 3 | Activity Group*Lesson Type | VPA | lsmeans

Activity Group	LessonType	lsmean	SE	df	lower.CL	upper.CL
Invasion games	Girls	0.0197	0.227	225.4	-0.427	0.4665
Net/wall/racket games	Girls	-0.0317	0.445	297	-0.907	0.8439
Fielding/striking games	Girls	-0.3135	0.197	205.8	-0.702	0.0747
Athletics	Girls	0.1261	0.316	280.3	-0.496	0.7484
Fitness	Girls	-0.5695	0.325	309.3	-1.21	0.0705
Adventure/Games	Girls	-0.5018	0.56	274.7	-1.604	0.6005
Various	Girls	-0.1548	0.214	226.5	-0.577	0.2673
Athletics-Field	Girls	-0.3095	0.342	303.2	-0.982	0.3635
Athletics-Track	Girls	nonEst	NA	NA	NA	NA
Invasion games	Boys	0.1838	0.223	193.2	-0.256	0.6239
Net/wall/racket games	Boys	-0.3942	0.27	292.4	-0.926	0.138
Fielding/striking games	Boys	-0.1589	0.19	152.7	-0.534	0.2156
Athletics	Boys	-0.0343	0.315	300.9	-0.654	0.5853
Fitness	Boys	0.3213	0.27	284.7	-0.211	0.8532
Adventure/Games	Boys	-0.7201	0.42	298.6	-1.547	0.1065
Various	Boys	-0.95	0.583	313.8	-2.097	0.1972
Athletics-Field	Boys	-1.1493	0.448	92.4	-2.039	-0.26
Athletics-Track	Boys	-0.1676	0.415	297.4	-0.984	0.6492
Invasion games	Mixed	-0.0955	0.274	316.9	-0.635	0.4439
Net/wall/racket games	Mixed	-0.4406	0.244	321.2	-0.921	0.0395
Fielding/striking games	Mixed	-0.6758	0.201	153.3	-1.073	-0.2785
Athletics	Mixed	0.0257	0.339	280.2	-0.642	0.6937
Fitness	Mixed	1.0863	0.334	299.8	0.43	1.7428
Adventure/Games	Mixed	-0.0203	0.556	269.6	-1.115	1.0744
Various	Mixed	-0.4141	0.174	138.6	-0.757	-0.0708
Athletics-Field	Mixed	-0.9618	0.335	238.3	-1.623	-0.3009
Athletics-Track	Mixed	0.3065	0.242	289.2	-0.169	0.7819

Exploring activity levels in physical education lessons in the UK: A cross-sectional examination of activity types and fitness levels

Table 3 | Model 2 | Activity Group | MVPA | lsmeans

Activity Group	lsmean	SE	df	lower.CL	upper.CL
Invasion games	0.341	0.149	216	0.047069	0.6356
Net/wall/racket games	0.174	0.159	316	-0.138372	0.4863
Fielding/striking games	-0.21	0.123	170	-0.452468	0.0334
Athletics	-0.102	0.189	278	-0.472863	0.2697
Fitness	0.566	0.19	275	0.192397	0.9391
Adventure/Games	-0.462	0.286	268	-1.024355	0.1006
Various	-0.125	0.125	165	-0.37155	0.1221
Athletics-Field	-0.823	0.208	214	-1.231889	-0.4137
Athletics-Track	0.367	0.186	386	0.000293	0.733

Table 4 | Model 3 | Activity Group*Lesson Type | MVPA | lsmeans

Activity Group	LessonType	lsmean	SE	df	lower.CL	upper.CL
Invasion games	Girls	0.2617	0.214	225	-0.1602	0.6835
Net/wall/racket games	Girls	0.9681	0.431	303	0.1193	1.8169
Fielding/striking games	Girls	-0.3982	0.185	207	-0.7634	-0.0329
Athletics	Girls	0.0418	0.302	280	-0.5526	0.6363
Fitness	Girls	-0.3185	0.313	312	-0.9337	0.2968
Adventure/Games	Girls	-0.5064	0.549	284	-1.5879	0.5752
Various	Girls	-0.0656	0.202	230	-0.4644	0.3332
Athletics-Field	Girls	-0.5817	0.33	305	-1.2312	0.0677
Athletics-Track	Girls	nonEst	NA	NA	NA	NA
Invasion games	Boys	0.3364	0.21	191	-0.0771	0.75
Net/wall/racket games	Boys	0.0554	0.258	292	-0.4531	0.5639
Fielding/striking games	Boys	-0.106	0.177	153	-0.4551	0.2432
Athletics	Boys	-0.2419	0.304	302	-0.84	0.3562
Fitness	Boys	0.7339	0.258	286	0.2262	1.2417
Adventure/Games	Boys	-0.7335	0.406	300	-1.5321	0.0651
Various	Boys	-0.9797	0.564	317	-2.0902	0.1309
Athletics-Field	Boys	-1.0823	0.41	91	-1.8963	-0.2682
Athletics-Track	Boys	-0.1661	0.401	298	-0.9556	0.6234
Invasion games	Mixed	0.2098	0.263	315	-0.3069	0.7264
Net/wall/racket games	Mixed	0.027	0.234	334	-0.4332	0.4873
Fielding/striking games	Mixed	-0.3039	0.188	152	-0.6746	0.0668
Athletics	Mixed	-0.1711	0.328	281	-0.8174	0.4752
Fitness	Mixed	0.7814	0.32	300	0.1508	1.4119
Adventure/Games	Mixed	-0.1641	0.539	272	-1.2259	0.8977
Various	Mixed	-0.2078	0.161	140	-0.5268	0.1111
Athletics-Field	Mixed	-1.0156	0.318	233	-1.6422	-0.389
Athletics-Track	Mixed	0.3189	0.23	306	-0.1344	0.7722

Table 5 | Model 2 | Activity Group | SPA | lsmeans

Activity Group	lsmean	SE	df	lower.CL	upper.CL
Invasion games	-0.387	0.146	212	-0.6741	-0.09922
Net/wall/racket games	-0.296	0.155	314	-0.6014	0.00885
Fielding/striking games	0.147	0.12	168	-0.0897	0.38343
Athletics	0.191	0.185	277	-0.1745	0.55577
Fitness	-0.572	0.186	273	-0.9383	-0.20529
Adventure/Games	0.309	0.282	268	-0.2463	0.86408
Various	0.149	0.122	163	-0.0915	0.38954
Athletics-Field	0.942	0.203	208	0.5423	1.34233
Athletics-Track	-0.153	0.182	382	-0.5113	0.20537

Exploring activity levels in physical education lessons in the UK: A cross-sectional examination of activity types and fitness levels

Table 6 | Model 3 | Activity Group*Lesson Type | SPA | lsmeans

Activity Group	LessonType	lsmean	SE	df	lower.CL	upper.CL
Invasion games	Girls	-0.37379	0.215	221.1	-0.7979	0.0503
Net/wall/racket games	Girls	-1.09657	0.435	301.4	-1.9517	-0.2414
Fielding/striking games	Girls	0.28178	0.186	203.8	-0.0854	0.6489
Athletics	Girls	-0.02647	0.304	275.8	-0.6243	0.5714
Fitness	Girls	-0.24808	0.315	308.1	-0.8672	0.371
Adventure/Games	Girls	0.1416	0.553	279.7	-0.9467	1.2299
Various	Girls	-0.01638	0.204	227.7	-0.4176	0.3848
Athletics-Field	Girls	0.72495	0.332	301.8	0.0708	1.3791
Athletics-Track	Girls	nonEst	NA	NA	NA	NA
Invasion games	Boys	-0.44331	0.211	188.2	-0.859	-0.0276
Net/wall/racket games	Boys	-0.29381	0.26	288	-0.8053	0.2177
Fielding/striking games	Boys	0.11637	0.177	150.1	-0.2342	0.467
Athletics	Boys	0.2544	0.306	299.9	-0.3481	0.8569
Fitness	Boys	-0.80308	0.26	282.9	-1.314	-0.2921
Adventure/Games	Boys	0.56927	0.409	297.7	-0.2353	1.3739
Various	Boys	1.15076	0.568	312.8	0.0331	2.2684
Athletics-Field	Boys	1.21921	0.41	89.2	0.4037	2.0347
Athletics-Track	Boys	0.01577	0.404	295.8	-0.7797	0.8113
Invasion games	Mixed	-0.12472	0.263	312.1	-0.6423	0.3929
Net/wall/racket games	Mixed	-0.04981	0.233	327.2	-0.5088	0.4092
Fielding/striking games	Mixed	0.22717	0.188	149.8	-0.1448	0.5992
Athletics	Mixed	0.41556	0.331	279.6	-0.2363	1.0674
Fitness	Mixed	-0.2882	0.323	297.3	-0.9233	0.3469
Adventure/Games	Mixed	-0.00435	0.544	271.7	-1.0759	1.0672
Various	Mixed	0.30931	0.162	138.6	-0.011	0.6296
Athletics-Field	Mixed	1.0559	0.32	229.5	0.4257	1.6861
Athletics-Track	Mixed	-0.05507	0.23	298.3	-0.5086	0.3985

Table 7 | Model 3 | Activity Group*Lesson Type | VPA (Girls only) | lsmeans

Activity Group	LessonType	lsmean	SE	df	lower.CL	upper.CL
Invasion games	Girls	0.313	0.413	104.6	-0.506	1.132
Athletics	Girls	0.2666	0.512	121.6	-0.746	1.28
Net/wall/racket games	Girls	0.104	0.495	121.1	-0.877	1.085
Athletics-Field	Girls	-0.0773	0.482	119.9	-1.032	0.878
Various	Girls	-0.0993	0.43	116.5	-0.952	0.753
Fielding/striking games	Girls	-0.1084	0.405	106.8	-0.911	0.694
Fitness	Girls	-0.3847	0.5	122.1	-1.373	0.604
Adventure/Games	Girls	-0.4194	0.669	105.7	-1.746	0.907
Fitness	Girls-Mixed	0.9499	0.987	82.3	-1.014	2.914
Adventure/Games	Girls-Mixed	0.5377	0.756	112.5	-0.959	2.035
Athletics	Girls-Mixed	0.4552	0.753	111.7	-1.037	1.947
Fielding/striking games	Girls-Mixed	-0.2247	0.508	120.8	-1.23	0.781
Various	Girls-Mixed	-0.4897	0.685	87.8	-1.851	0.871
Net/wall/racket games	Girls-Mixed	-0.4505	0.548	125.2	-1.536	0.635
Invasion games	Girls-Mixed	-0.5778	1.033	108.2	-2.625	1.47
Athletics-Field	Girls-Mixed	nonEst	NA	NA	NA	NA
