# Supplementary material for: Exploring activity levels in physical education lessons in the UK: a cross-sectional examination of activity types and fitness levels
Source: BMJ Open Sport Exerc Med. 2021 Mar 9;7(1):e000924. doi: 10.1136/bmjsem-2020-000924 (PMC7944978; doi:10.1136/bmjsem-2020-000924)
Supplement: Supplementary data [file bmjsem-2020-000924supp012.pdf]

Exploring activity levels in physical education lessons in the UK: A cross-sectional examination of activity types and fitness levels

SUPPLEMENTARY FILE 12: Post-hoc LSMeans tables

Tables: Post-hoc LS Means, Contrasts and significances for Model 2 & 3, and SPA, VPA and MVPA

Model 2: Activity Group Main Effect				VPA	SPA	MVPA
				Yes	Yes	Yes
	Invasion Games (+)		Fielding/striking games (-)	0.492**	-0.538***	0.554***
	Invasion Games (+)		Athletics-Field (-)	0.808*	-1.339***	1.168***
	Invasion Games (+)		Various (-)		-0.548*	
	Net/wall/racket games(+)		Fielding/striking games (-)		-0.442*	
	Net/wall/racket games (+)		Athletics-Field (-)		-1.241***	1.003***
	Net/wall/racket games (-)		Athletics-Track (+)	-0.754**		
	Net/wall/racket games (-)		Fitness (+)	-0.663*		
	Fielding/striking games (+)		Athletics-Field (-)		-0.800**	
	Fielding/striking games (-)		Athletics-Track (+)	-0.868***		-0.574***
	Fielding/striking games (-)		Fitness (+)	-0.777***	0.720***	-0.778***
	Athletics (-)		Fitness (+)		0.764*	
	Athletics-Field (-)		Athletics-Track (+)	-1.185***	1.098***	-1.188***
	Fitness (+)		Athletics-Field (-)	1.094**	-1.520***	1.391***
	Fitness (+)		Adventure/Games (-)			1.031*
	Fitness (+)		Various (-)	0.684*	-0.730**	0.695*
	Various (+)		Athletics-Field (-)		-0.790**	0.697*
	Various (-)		Athletics-Track (+)	-0.775**		
**Model 3: Activity Group*Lesson Type Interaction Effect**				**Yes**	**Yes**	**Yes**
Mixed	Net/wall/racket games (-)	Mixed	Fitness (+)	-1.542**		
Mixed	Fielding/striking games (-)	Mixed	Athletics-Track (+)	-0.992***		-0.636**
Mixed	Fielding/striking games (-)	Mixed	Fitness (+)	-1.769***		
Mixed	Athletics-Field (-)	Mixed	Athletics-Track (+)	-1.286*		-1.353**
Mixed	Fitness (+)	Mixed	Athletics-Field (-)	2.063***		1.805**
Mixed	Fitness (+)	Mixed	Various (-)	1.510***		
Boys	Invasion Games (+)	Boys	Athletics-Field (-)		-1.660*	
Boys	Fitness (+)	Boys	Athletics-Field (-)		-2.016**	1.810*
Boys	Invasion Games (+)	Mixed	Fielding/striking games (-)	0.853**		
Boys	Fitness (+)	Mixed	Fielding/striking games (-)	0.984*	-1.007*	1.022**
Boys	Invasion Games (+)	Mixed	Athletics-Field (-)		-1.491**	1.348*
Boys	Fitness (+)	Mixed	Athletics-Field (-)		-1.846***	1.739***
Boys	Net/wall/racket games (-)	Mixed	Fitness (+)	-1.494*		
Boys	Fielding/striking games (-)	Mixed	Fitness (+)	-1.256*		
Boys	Athletics-Field (-)	Mixed	Fitness (+)	-2.252**		-1.876*
Boys	Invasion Games (+)	Mixed	Various (-)		-0.743*	
Boys	Fitness (+)	Mixed	Various (-)		-1.099**	
Girls	Net/wall/racket games (+)	Girls	Fielding/striking games (-)			
Girls	Net/wall/racket games (+)	Girls	Athletics-Field (-)			
Girls	Fitness (-)	Girls	Athletics-Field (+)			
Girls	Net/wall/racket games (+)	Mixed	Athletics-Field (-)			
Girls	Fitness (-)	Mixed	Fitness (+)	-1.732*		
Girls	Fielding/striking games (-)	Boys	Fitness (+)			
Girls	Athletics-Field (-)	Boys	Fitness (+)		1.622*	

Notes

- The direction of the effect is indicated by a (+) for ‘more active’, and a (-) for ‘less active’.
- *** p<.001; ** p<.0125; * p <.05. Values that are inferior to 0.0125 are highlighted in red.
