# Supplementary material for: Exploring activity levels in physical education lessons in the UK: a cross-sectional examination of activity types and fitness levels
Source: BMJ Open Sport Exerc Med. 2021 Mar 9;7(1):e000924. doi: 10.1136/bmjsem-2020-000924 (PMC7944978; doi:10.1136/bmjsem-2020-000924)
Supplement: Supplementary data [file bmjsem-2020-000924supp013.pdf]

Exploring activity levels in physical education lessons in the UK: A cross-sectional examination of activity types and fitness levels

SUPPLEMENTARY FILE 13: Lesson Location

When considering factors affecting PA, a recent systematic review[1] identified modifiable variables that were consistently associated with levels of MVPA in PE including the class sex, the type of PE activities and content, **lesson location (outdoors)**, beliefs and values of students, and enjoyment of exercise.

RESULTS

Table: Pupil average time (%) in PE lessons split by PA domain, for lesson location, at the A) Lesson-level and B) Pupil-level

	A) Lesson-level				B) Pupil-level			
**% of 1hr Lesson (SD)**	**Indoors (n=49)**	**Outdoors (n=177)**	**In/Out (n=23)**	**Overall (n=249)**	**Indoors (n=1565)**	**Outdoors (n=6415)**	**In/Out (n=1503)**	**Overall (n=9483)**
**PA Domain**								
SPA	44.5 (14.3)	43.0 (12.2)	44.5 (13.1)	44.3 (8.93)	47.6 (15.6)	43.7 (12.8)	42.6 (12.2)	44.2 (13.3)
LPA	32.2 (7.82)	32.9 (7.23)	31.4 (7.76)	32.2 (4.43)	28.9 (8.53)	32.5 (7.35)	33.2 (7.33)	32.0 (7.68)
MPA	16.6 (6.72)	17.0 (6.07)	16.6 (6.49)	16.7 (4.28)	16.3 (7.64)	16.6 (6.23)	17.4 (6.07)	16.7 (6.47)
VPA	6.68 (4.56)	7.12 (4.09)	7.44 (4.54)	7.00 (2.98)	7.20 (5.26)	7.21 (4.33)	6.79 (4.06)	7.14 (4.46)
MVPA	23.3 (10.1)	24.1 (8.83)	24.1 (9.24)	23.7 (6.27)	23.5 (10.9)	23.8 (9.13)	24.2 (8.93)	23.8 (9.42)

For MVPA and VPA, similar PA levels were recorded for lesson locations (23.9% v 23.6%, and 7.0% vs 7.1% for indoor vs outdoor lessons respectively).

Multi-level Models of Physical Activity Levels during PE

The following tables summarise associations between PA levels and predictor variables. The interaction of **'activity group by lesson location'** was examined. As summarized (Table 1 | Model estimates - SPA), outdoor lessons were less sedentary than indoor lessons. For MVPA, no significant differences were observed for the interaction (Table 1 | Model estimates - MVPA). Post-hoc analysis (Table 4 - VPA) showed that showed outdoor track athletics and invasion games were more vigorously active than indoor fielding/striking games and outdoor field athletics. For SPA (Table 4 - SPA), regardless of location, fielding/striking games and field athletics were more sedentary than outdoor invasion games, and net/wall/racket games and track athletics respectively. Outdoor field athletics was more sedentary than indoor fitness, and outdoor fielding/striking games.

Exploring activity levels in physical education lessons in the UK: A cross-sectional examination of activity types and fitness levels

Table 1: Summary of PA in PE model estimates, CIs and p-values

	VPA		MVPA		SPA	
**Model estimate** [a]	**ß**	**95% CI**	**ß**	**95% CI**	**ß**	**95% CI**
(Intercept)	0.18	-0.48 to 0.84	0.20	-0.41 to 0.81	0.04	-0.54 to 0.62
Net/wall/racket games[b]	-0.43	-0.93 to 0.07	-0.11	-0.60 to 0.38	-0.16	-0.65 to 0.32
Fielding/striking games[b]	-0.86***	-1.32 to -0.40	-0.80***	-1.24 to -0.36	0.39	-0.05 to 0.82
Athletics	-0.06	-0.44 to 0.32	-0.45*	-0.81 to -0.08	0.63***	0.27 to 0.99
Fitness[b]	0.24	-0.19 to 0.66	0.18	-0.23 to 0.59	-0.31	-0.72 to 0.09
Adventure/Games[b]	-1.05**	-1.87 to -0.24	-1.21**	-2.00 to -0.42	0.94*	0.16 to 1.72
Various	-0.58	-1.28 to 0.12	-0.75*	-1.42 to -0.09	0.76*	0.11 to 1.42
Athletics-Field[b]	-0.18	-0.83 to 0.47	-0.76*	-1.38 to -0.14	0.78*	0.17 to 1.39
Athletics-Track[b]	0.17	-0.21 to 0.55	-0.12	-0.49 to 0.25	0.28	-0.08 to 0.64
Location-Outdoors[c]	0.35	-0.03 to 0.72	0.33	-0.03 to 0.70	-0.71***	-1.07 to -0.35
ActivityGroupNet/wall/racket games:LocationOutdoors	-0.00	-0.60 to 0.60	-0.19	-0.77 to 0.40	0.43	-0.15 – 1.00
ActivityGroupFielding/striking games:LocationOutdoors	0.47	-0.01 to 0.95	0.30	-0.17 to 0.77	0.19	-0.28 – 0.65
ActivityGroupFitness:LocationOutdoors	-0.41	-1.55 to 0.73	-0.26	-1.35 to 0.84	0.32	-0.75 – 1.39
ActivityGroupAdventure/Games:LocationOutdoors	0.94	-0.16 to 2.04	0.70	-0.36 to 1.76	-0.50	-1.55 – 0.54
ActivityGroupVarious:LocationOutdoors	0.18	-0.55 to 0.92	0.27	-0.43 to 0.98	-0.16	-0.85 – 0.52
ActivityGroupAthletics-Field:LocationOutdoors	-0.91*	-1.63 to -0.20	-0.58	-1.28 to 0.11	0.77*	0.08 – 1.45

[a] Fully-adjusted model including lesson length, lesson type, and school effects; SPA/MVPA are orderNorm transformed; VPA are Yeo-Johnson transformed.
[b] Reference category: Invasion games; [c] Reference category: Indoors
[d] Reference category: ActivityGroupInvasion games:LocationIndoors
*** p<.001; ** p<.0125; * p <.05
Significant differences observed for ActivityGroup*LessonType for VPA and SPA; post-hoc analysis conducted.

Notes

*Model (ActivityGroup*Location of PE lesson)* This model was developed to explore the interaction of activity and PE Lesson location.

Exploring activity levels in physical education lessons in the UK: A cross-sectional examination of activity types and fitness levels

Tables: summary of the fixed effect interactions (LS Means) for Model 4, for VPA, MVPA & SPA

Table 2 | Model | Activity Group*Lesson Location | VPA | lsmeans

Activity Group	Location	lsmean	SE	df	lower.CL	upper.CL
Invasion games	Indoors	-0.1064	0.221	257	-0.5413	0.3285
Net/wall/racket games	Indoors	-0.5327	0.218	403	-0.962	-0.1035
Fielding/striking games	Indoors	-0.9593	0.188	294	-1.3296	-0.5891
Athletics	Indoors	nonEst	NA	NA	NA	NA
Fitness	Indoors	0.1325	0.192	243	-0.2452	0.5101
Adventure/Games	Indoors	-1.1535	0.409	253	-1.9585	-0.3485
Various	Indoors	-0.679	0.327	248	-1.3224	-0.0355
Athletics-Field	Indoors	-0.2813	0.31	269	-0.8922	0.3296
Athletics-Track	Indoors	nonEst	NA	NA	NA	NA
Invasion games	Outdoors	0.2442	0.165	206	-0.0814	0.5699
Net/wall/racket games	Outdoors	-0.1902	0.201	320	-0.5848	0.2044
Fielding/striking games	Outdoors	-0.1414	0.136	145	-0.4108	0.128
Athletics	Outdoors	0.1887	0.196	258	-0.1976	0.5749
Fitness	Outdoors	0.0785	0.582	242	-1.0686	1.2257
Adventure/Games	Outdoors	0.1355	0.397	222	-0.6467	0.9178
Various	Outdoors	-0.1457	0.168	165	-0.4776	0.1861
Athletics-Field	Outdoors	-0.8465	0.247	225	-1.3323	-0.3606
Athletics-Track	Outdoors	0.4176	0.196	333	0.0313	0.8039

Model 4 | Activity Group*Lesson Location | MVPA
The interaction effect was not significant.

Table 3 | Model | Activity Group*Lesson Location | SPA | lsmeans

Activity Group	Location	lsmean	SE	df	lower.CL	upper.CL
Invasion games	Indoors	0.14284	0.206	257	-0.262	0.548
Net/wall/racket games	Indoors	-0.02937	0.204	398	-0.431	0.372
Fielding/striking games	Indoors	0.52101	0.173	301	0.181	0.8613
Athletics	Indoors	nonEst	NA	NA	NA	NA
Fitness	Indoors	-0.17726	0.175	245	-0.523	0.1681
Adventure/Games	Indoors	1.07449	0.388	261	0.311	1.838
Various	Indoors	0.89613	0.3	239	0.305	1.4877
Athletics-Field	Indoors	0.91557	0.292	275	0.341	1.4898
Athletics-Track	Indoors	nonEst	NA	NA	NA	NA
Invasion games	Outdoors	-0.56981	0.149	202	-0.863	-0.2766
Net/wall/racket games	Outdoors	-0.31401	0.187	321	-0.682	0.0535
Fielding/striking games	Outdoors	0.00343	0.12	142	-0.234	0.2407
Athletics	Outdoors	0.05543	0.181	259	-0.301	0.4121
Fitness	Outdoors	-0.56815	0.549	248	-1.649	0.5131
Adventure/Games	Outdoors	-0.13935	0.378	230	-0.885	0.6059
Various	Outdoors	0.02164	0.15	164	-0.274	0.3169
Athletics-Field	Outdoors	0.97543	0.224	210	0.534	1.4168
Athletics-Track	Outdoors	-0.28991	0.182	345	-0.647	0.0675

Exploring activity levels in physical education lessons in the UK: A cross-sectional examination of activity types and fitness levels

Table 4: Post-hoc LS Means, Contrasts and significances, and SPA, VPA and MVPA

Model: Activity Group*Location Interaction Effect				VPA	SPA	MVPA
				**Yes**	**Yes**	**No**
Indoor	Invasion Games (-)	Outdoor	Invasion Games (+)		0.707*	
Indoor	Invasion Games (-)	Outdoor	Net/wall/racket games (+)			
Indoor	Net/wall/racket games (+)	Outdoor	Athletics-Field (-)		-1.005*	
Indoor	Net/wall/racket games (-)	Outdoor	Athletics-Track (+)	-0.949**		
Indoor	Fielding/striking games (-)	Outdoor	Invasion Games (+)	-1.208***	1.094***	
Indoor	Fielding/striking games (-)	Outdoor	Net/wall/racket games (+)		0.832*	
Indoor	Fielding/striking games (-)	Outdoor	Fielding/striking games (+)	-0.820***		
Indoor	Fielding/striking games (-)	Outdoor	Athletics (+)	-1.150***		
Indoor	Fielding/striking games (-)	Outdoor	Athletics-Track (+)	-1.377***	0.811***	
Indoor	Fielding/striking games (-)	Outdoor	Various (+)	-0.811*		
Indoor	Athletics-Field (-)	Outdoor	Invasion Games (+)		1.488***	
Indoor	Athletics-Field (-)	Outdoor	Net/wall/racket games (+)		1.226*	
Indoor	Athletics-Field (-)	Outdoor	Fielding/striking games (+)			
Indoor	Athletics-Field (-)	Outdoor	Athletics-Track (+)		1.205*	
Indoor	Fitness (+)	Outdoor	Athletics-Field (-)		-1.156**	
Indoor	Adventure/Games (-)	Outdoor	Invasion Games (+)		1.645**	
Indoor	Adventure/Games (-)	Outdoor	Athletics-Track (+)	-1.570*		
Indoor	Various (-)	Outdoor	Invasion Games (+)		1.470**	
Indoor	Various (-)	Outdoor	Net/wall/racket games (+)			
Indoor	Various (-)	Outdoor	Fielding/striking games (+)			
Indoor	Various (-)	Outdoor	Athletics-Track (+)			
Indoor	Various (-)	Outdoor	Various (+)			
Outdoor	Invasion Games (+)	Outdoor	Fielding/striking games (-)		-0.572**	
Outdoor	Invasion Games (+)	Outdoor	Athletics-Field (-)	1.094**	-1.548***	
Outdoor	Net/wall/racket games (+)	Outdoor	Athletics-Field (-)		-1.286***	
Outdoor	Fielding/striking games (+)	Outdoor	Athletics-Field (-)		-0.974**	
Outdoor	Athletics (+)	Outdoor	Athletics-Field (-)	1.037*		
Outdoor	Athletics-Field (-)	Outdoor	Athletics-Track (+)	-1.263***	1.265***	
Outdoor	Various (+)	Outdoor	Athletics-Field (-)		-0.948*	

Notes
- The direction of the effect is indicated by a (+) for 'more active', and a (-) for 'less active'.
- *** p<.001; ** p<.0125; * p <.05. Values that are inferior to 0.0125 are highlighted in red.

DISCUSSION

In general, we found outdoor lessons to be more vigorously active, and significantly less sedentary, than indoor lessons, apart from outdoor field athletics which was more sedentary than indoor fitness and indoor net/wall/racket games. Previous studies suggest that lesson location influences the intensity of PE lessons[2, 3] with a greater proportion of MVPA and VPA in outdoors compared to indoor classes, and less time spent doing SPA.

REFERENCES

1. Zhou Y, Wang L. Correlates of Physical Activity of Students in Secondary School Physical Education: A Systematic Review of Literature. *Biomed Res Int.* 2019;**2019**:1-12.
2. Chow BC, McKenzie TL, Louie, L. Physical Activity and Environmental Influences during Secondary School Physical Education. *J Teach Phys Educ.*2009;**28**:21-37.
3. McKenzie T, Catellier D, Conway T, *et a*l. Girls' activity levels and lesson contexts in middle school PE: TAAG baseline. *Med Sci Sports Exerc.*2006;**38**:1229-35.
